# The Efficacy and Safety of Chinese Herbal Medicine in the Treatment of Knee Osteoarthritis: An Updated Systematic Review and Meta-Analysis of 56 Randomized Controlled Trials

**DOI:** 10.1155/2022/6887988

**Published:** 2022-01-07

**Authors:** Zhou Lin, Junju Zheng, Mangmang Chen, Jiaru Chen, Jiejun Lin

**Affiliations:** ^1^Department of Orthopaedic Surgery, Wenzhou Center Hospital, Wenzhou, China; ^2^Department of Gastroenterology, Wenzhou Center Hospital, Wenzhou, China

## Abstract

**Objective:**

This systematic review and meta-analysis were performed to investigate the efficacy and safety of Chinese herbal medicine (CHM) in the treatment of knee osteoarthritis (KOA).

**Methods:**

An electronic search was conducted in eight databases (PubMed, EMBASE, Web of Science, Cochrane Library, Chinese National Knowledge Infrastructure, Chinese Biomedical Literature Database, Chinese VIP Database, and Wanfang Database) from inception until December 2019. The risk of bias assessment of the included RCTs was evaluated by Cochrane collaboration's tool. The inclusion criteria were RCTs that investigated the efficacy and safety of CHM in the treatment of KOA, with no restrictions on publication status or language. The exclusion criteria included nonrandomized or quasi-RCTs, no clear KOA diagnostic approach, combined Chinese medicinal herbs with other traditional Chinese medicine treatment modalities, and published using repeated data and missing data. We computed the relative risk (RR) and the standard mean difference (SMD) for dichotomous outcomes and continuous outcomes, respectively. When heterogeneity was detected or there was significant statistical heterogeneity (*P* < 0.05 or *I*^2^ > 50%), a random-effects model was employed, followed by further subgroup analysis and metaregression estimations to ascertain the origins of heterogeneity. Otherwise, we used a fixed-effects model (*P* ≥ 0.05 or *I*^2^ ≤ 50%). The primary outcome measures were visual analog score (VAS), Western Ontario and McMaster Universities Osteoarthritis Index (WOMAC), Lysholm score, and Lequesne index. Secondary outcome measures were the total clinical effective rate and adverse events. The meta-analysis was performed using the Stata 14.0 software.

**Results:**

A total of 56 RCTs comprising 5350 patients met the inclusion criteria. This meta-analysis showed that application of CHM as adjuvant therapy or monotherapy for KOA can significantly decrease VAS, WOMAC, and the Lequesne index and improve the Lysholm score as well as the total effective rate. In addition, this treatment has fewer adverse effects, suggesting that CHM is generally safe and well tolerated among patients with KOA.

**Conclusion:**

Our study offers supportive evidence that CHM, either adjuvant therapy or monotherapy, reduces the VAS, WOMAC, and Lequesne index and improves the Lysholm score and overall effective rate in patients with KOA. Additionally, CHM was well tolerated and safe in KOA patients. We found frequently used CHMs that might contribute to the formulation of a herbal formula that could be considered for further clinical use. However, given the heterogeneity and limited sample size in this study, larger multicenter and high-quality RCTs are needed to validate the benefits of CHM in the treatment of KOA.

## 1. Introduction

Knee osteoarthritis (KOA) is a multifactorial degenerative joint disorder characterized by changes in the structure of the joint tissues, including cartilage degeneration, subchondral bone restructuring, and synovial membrane inflammation in the elderly [1]. KOA is more prevalent in older adults [2]. A previous study reported that approximately 12% of the aging population in the West suffered from KOA, and 25% of the population above 55 years old had a persistent knee pain episode [3]. According to current data, 9.3 million adults in the US are affected by KOA [4]. As the population ages, it is projected that the number of persons with KOA will increase [5, 6]. Osteoarthritis was projected to become the fourth leading cause of disability by 2021 [7].

The primary management goals for KOA have been to alleviate pain, educate patients about the disease, rehabilitate, slow the progression of the disease, and maintain a healthy lifestyle [8]. However, effective therapeutic strategies for KOA disease modification are currently unavailable [9]. The current therapeutic options advanced in various evidence-based clinical guidelines include nonpharmacological therapies, weight loss, oral pharmacological medications, exercise, topical therapies, surgical treatments, and intra-articular therapies [10–12]. Notably, nonsteroidal anti-inflammatory drugs (NSAIDs) and intra-articular hyaluronic acid or corticosteroids are the most frequently used in clinical practice [13–15]. Long-term use of NSAIDs and corticosteroids, on the other hand, has serious adverse effects [16]. Therefore, clinicians and patients are increasingly preferring to treat KOA using complementary as well as alternative medicine [17–19].

Chinese herbal medicine (CHM) has been used in various forms in the treatment of KOA, both in China and the rest of the world [20, 21]. The adoption of CHM for treating pain disorders, including KOA, has been steadily increasing in Asian countries as well as across the globe [22]. In comparison to other herbal medicines, CHM contains distinct medicinal components that target specific biological processes associated with disease, which are dependent on the differentiation of specific symptoms [23, 24]. According to a recent study, CHM actively reduces pain via analgesic, invigorating blood circulation, and anti-inflammatory effects [25].

CHM has long been regarded as a vital component in the treatment of KOA in China and is gaining popularity in other parts of the world. However, quantitative research evidence on its effects is currently limited. CHM's biological effect and potential interactions with other prescription medications have not yet been elucidated [26]. Two systematic reviews found that CMH is both safe and effective in the treatment of KOA [27, 28]. However, due to the low quality of the methodology and the limited sample size in the included studies, there is a knowledge gap on the planned application of CHM in treating KOA. Recently, there has been an increase in the number of high-quality randomized controlled clinical trials (RCTs) on the safety and efficacy of CHM in the treatment of KOA. Therefore, we conducted a large sample size systematic review and meta-analysis of high-quality RCTs focusing on CHM for treating KOA, excluding low-quality studies, in accordance with Cochrane's group guidelines for clinical reviews [29].

## 2. Methods

We used the PRISMA (Preferred Reporting Items for Systematic Reviews and Meta-Analyses) Statement to perform a systematic review and meta-analysis [30]. This study has been registered at http://www.researchregistry.com, and the study's unique identifying number (UIN) from the Research Registry is reviewregistry971. There are no protocols preregistered for this review. We did not collect any primary personal data; hence, we did not require ethical approval.

### 2.1. Database and Search Strategies

We performed electronic searches in eight repositories from their inception to December 2019: PubMed, Web of Science, EMBASE, Cochrane Library, Chinese Biomedical Literature Database, Chinese National Knowledge Infrastructure, Wanfang Database, and Chinese VIP Database. Additionally, we performed manual searches in the references section of previously published systematic reviews for additional relevant literature. Moreover, the literature search was not limited to any language of publishing. The search criteria used for PubMed were provided as a supplementary material (available here) and were appropriately modified for the other databases.

### 2.2. Eligibility Criteria

#### 2.2.1. Types of Studies

We included RCTs that investigated the efficacy and safety of CHM in the treatment of KOA, with no restrictions on publication status or language. If we discovered a relevant study with three treatment arms, we only retrieved data for the CHM arm(s) and the control arm(s). We excluded quasirandomized trials, such as studies in which subjects were allocated based on their date of birth, as well as the order in which they were admitted.

#### 2.2.2. Participant Types

We included subjects diagnosed with KOA based on the Chinese Orthopedic Association (COA) criteria (2007), American College of Rheumatology (ACR) criteria (1986 or 1995), and Chinese Rheumatology Association (CRA) criteria (2003, 2005, or 2010) regardless of disease course and severity, age, or gender.

#### 2.2.3. Types of Interventions

Regardless of the dosage, duration, administration route, administration techniques, or duration of therapy, the evaluated therapeutic intervention constituted CHM as monotherapy or a complement to western conventional medicine (WCM). The control group received WCM either alone or in combination with placebo. We excluded trials in which multiple types of CHM therapies were compared.

#### 2.2.4. Outcome Measure Types

The primary outcome parameters included the following: (1) visual analog score (VAS), (2) Western Ontario and McMaster Universities Osteoarthritis Index (WOMAC), (3) Lysholm score, and (4) Lequesne index. The secondary outcome parameters included the following: (1) the overall clinical effectiveness rate and (2) adverse events.

### 2.3. Literature Selection

The PRISMA flow diagram was used to select the trials that were included. We imported the literature results into the Endnote X7 software. Two independent authors initially screened the titles and abstracts of potentially eligible articles to remove duplications as well as RCTs that did not meet the inclusion criteria. Following that, we downloaded and reviewed the full texts of the remaining prospective studies. Any disagreements between the two authors were resolved through discussion with a third independent author.

### 2.4. Data Extraction

Two independent reviewers extracted the data, while a third independent reviewer checked for consistency. A standard form was used to collect the retrieved items, which included the following basic research information: the name(s) of the author(s), publication date, study design, diagnostic criteria, sample size, age, CHM and WCM intervention methods, gender, disease duration, and course of treatment. We retrieved the mean, standard deviation (SD), and the number of participants in each study for continuous outcomes. For dichotomous outcomes, we retrieved the total number of CHM and WCM events as well as the number of occurrences in each group. Where possible, we recomputed the data in other formats to allow for pooled analysis. Any disagreements that arose between these two reviewers were resolved through dialogue. We contacted the relevant authors of the included studies to provide us with any missing data and additional information.

### 2.5. Quality Assessment of Included Studies

Two independent authors used the Cochrane collaboration tool to assess the quality of methodology and risk of bias of the included RCT studies [31]. This Cochrane tool assesses the following parameters, randomization, subject blinding, allocation concealment, outcome evaluation blinding, selective outcome reporting, incomplete outcome data, and other bias, and categorizes studies as unclear, low, or high risk of bias for each item.

### 2.6. CHM Composition

We compiled a list of the major components of the CHM formulae. We determined the frequency of use of all Chinese medicinal herbs and estimated and discussed in detail those that were frequently used.

### 2.7. Statistical Analysis

The Stata software (version 12.0; StataCorp, College Station, TX) was used to evaluate all of the data retrieved in this study for meta-analysis. When heterogeneity was detected or there was significant statistical heterogeneity (*P* < 0.05 or *I*^2^ > 50%), a random-effects model was employed, followed by further subgroup analysis and metaregression estimations to ascertain the origins of heterogeneity. Otherwise, we used a fixed-effects model (*P* ≥ 0.05 or *I*^2^ ≤ 50%). We conducted a sensitivity analysis by excluding individual studies one by one to determine the strength and stability of the pooled data. Besides, the effect of publication bias was examined using Begg's and Egger's tests. We computed the relative risk (RR) and the standard mean difference (SMD) for dichotomous outcomes and continuous outcomes, respectively.

## 3. Results

### 3.1. Description of Studies

We identified 1532 potentially relevant hits from the repositories. After eliminating duplicated RCTs, we were left with 1241 peer-reviewed articles. Subsequently, we applied the inclusion and exclusion criteria to the titles and abstracts and eliminated 1114 irrelevant studies. Additionally, after reviewing the full text of the 127 remaining studies, we eliminated 71 studies for failing to meet at least one of the following criteria:

1. Nonrandomized or quasi-RCTs
2. No clear KOA diagnostic approach
3. Combined Chinese medicinal herbs with other TCM treatment modalities
4. Published using repeated data
5. Missing data.

**Figure 1:**
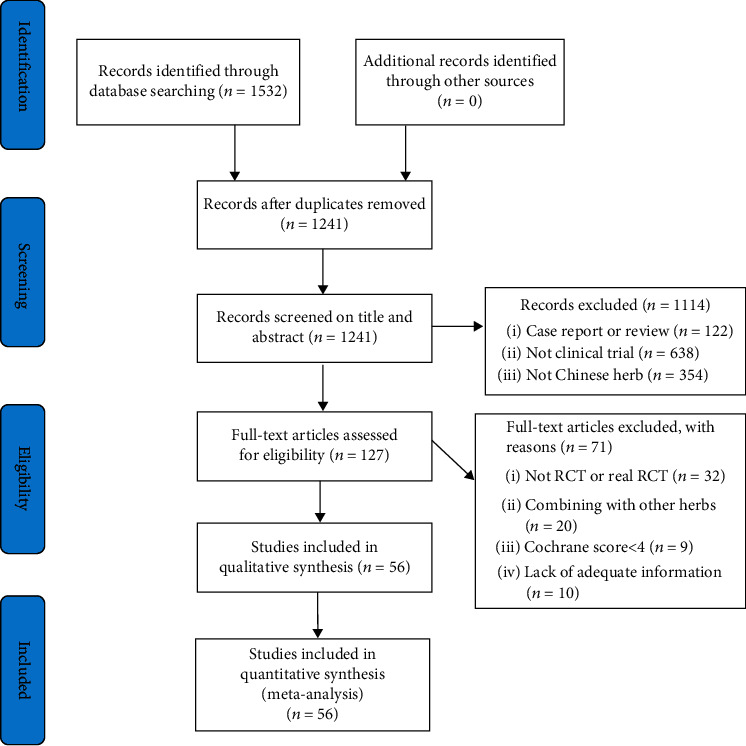
Flowchart of study selection.

Finally, 56 articles [32–87] were included for analysis (Figure 1).

### 3.2. Characteristics and Quality Assessment of the Eligible Studies

Table 1 summarizes the characteristics of the 56 included RCTs. All the included RCTs were published between 2009 and 2019. All studies enrolled 5350 patients, with 2710 in the treatment arm vs. 2640 in the control arm.  Table 2 summarizes the risk of bias analysis conducted on RCTs.  Table 3 summarizes the ingredients of CHM used in the included studies.

### 3.3. Results of Meta-Analysis

#### 3.3.1. VAS

**Figure 2:**
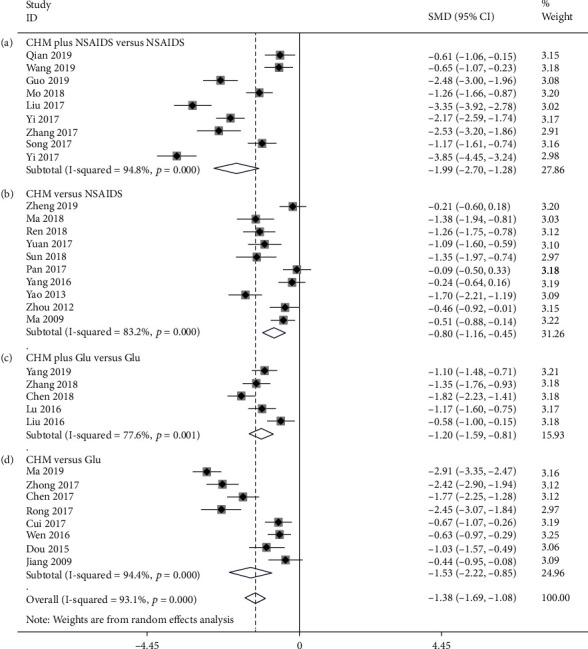
Forest plot of VAS: (a) CHM plus NSAIDS versus NSAIDS. (b) CHM versus NSAIDS. (c) CHM plus Glu versus Glu. (d) CHM versus Glu.

**Figure 3:**
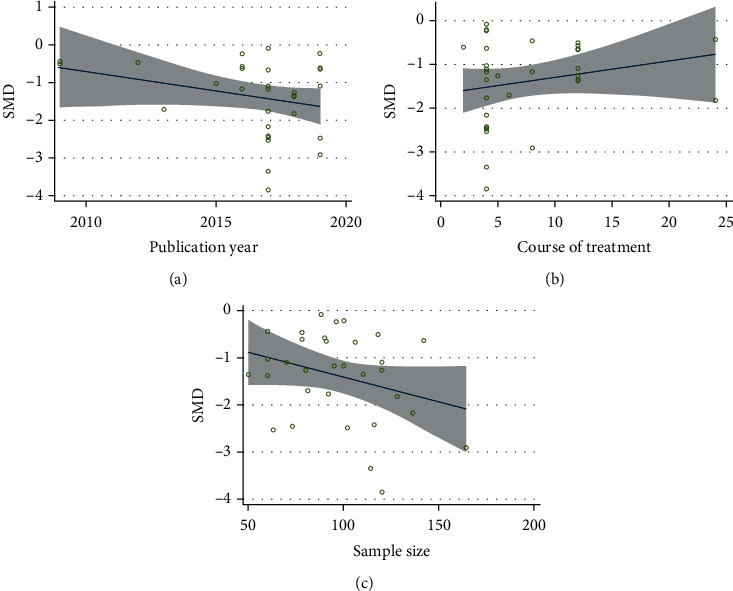
Metaregression analysis of VAS for (a) publication year, (b) course of treatment, (c) and sample size.

On the basis of regarding the VAS, nine studies compared CHM plus NSAIDS with NSAIDS alone [38, 46, 53, 55, 60, 65, 75, 81]. The pooled results revealed that CHM plus NSAIDS significantly decreased VAS when compared to NSAIDS alone (SMD = −1.990; 95%CI = −2.698 to − 1.282; *P* ≤ 0.001; heterogeneity *χ*^2^ = 155.33, df = 8, *I*^2^ = 94.8%, *P* ≤ 0.001) (Figure 2(a)). Ten studies compared the VAS of CHM and NSAIDS [50, 52, 54, 56, 61, 71, 73, 77, 83, 85]. The pooled results indicated that CHM monotherapy significantly reduced VAS when compared to NSAIDS alone (SMD = −0.803; 95%CI = −1.158 to − 0.449; *P* ≤ 0.001; heterogeneity *χ*^2^ = 53.68, df = 9, *I*^2^ = 83.2%, *P* ≤ 0.001, Figure 2(b)). We identified five studies that compared CHM plus Glu to Glu alone in terms of the VAS [33, 45, 48, 72, 80]. CHM plus Glu significantly decreased the VAS in comparison to Glu alone (SMD = −1.204; 95%CI = −1.593 to − 0.815; *P* = 0.001; heterogeneity *χ*^2^ = 17.89, df = 4, *I*^2^ = 77.6%, *P* = 0.001, Figure 2(c)). Eight studies compared CHM and Glu on the basis of their VAS scores [32, 35, 36, 43, 51, 58, 66, 84]. The pooled results indicated that CHM monotherapy significantly decreased VAS compared with Glu alone (SMD = −1.533; 95%CI = −1.688 to − 1.076; *P* ≤ 0.001; heterogeneity *χ*^2^ = 123.94, df = 7, *I*^2^ = 94.4%, *P* ≤ 0.001, Figure 2(d)). Metaregression was used to investigate the sources of heterogeneity in the findings. To ascertain the potential sources of interstudy heterogeneity, we conducted a metaregression analysis on the year of publication, course of treatment, and sample size (Figure 3). In general, the year of publication (*β* = −0.103; *P* = 0.120; *R*^2^ = 5.20%), the duration of treatment (*β* = 0.037; *P* = 0.231; *R*^2^ = 1.33%), and the sample size (*β* = −0.011; *P* = 0.100; *R*^2^ = 6.37%),were not significant sources of heterogeneity for the VAS.

#### 3.3.2. WOMAC

**Figure 4:**
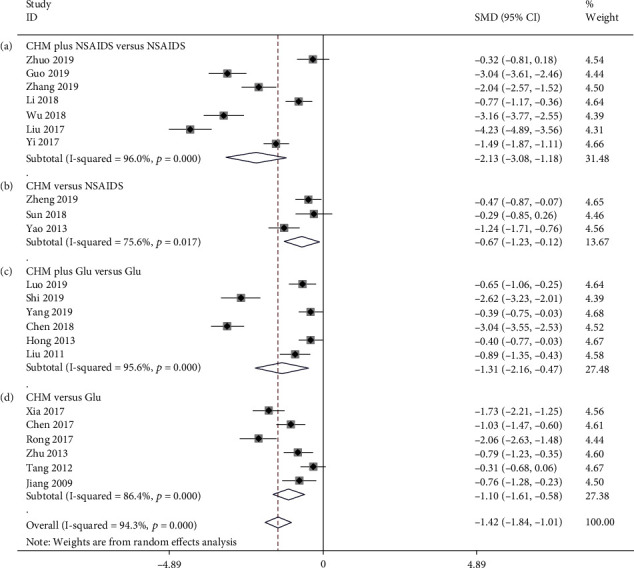
Forest plot of WOMAC: (a) CHM plus NSAIDS versus NSAIDS, (b) CHM versus NSAIDS, (c) CHM plus Glu versus Glu, and (d) CHM versus Glu.

**Figure 5:**
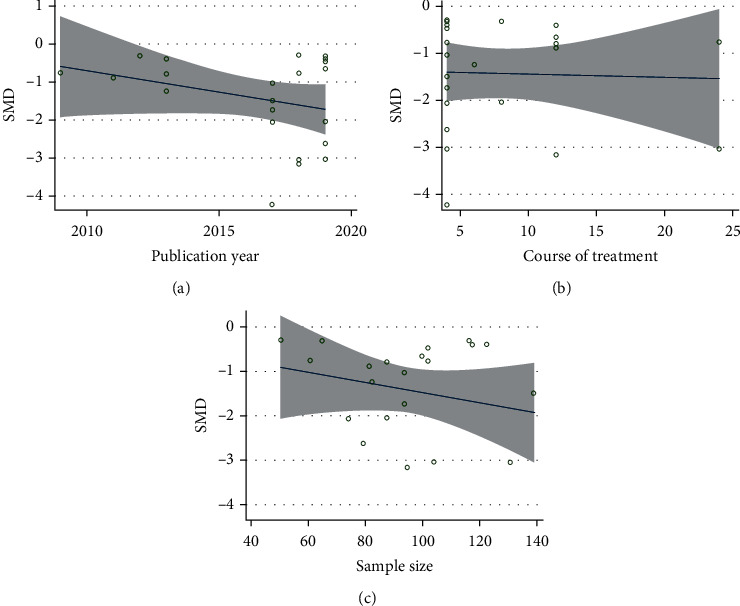
Metaregression analysis of WOMAC for (a) publication year, (b) course of treatment, (c) and sample size.

Seven studies compared CHM plus NSAIDS to NSAIDS alone in terms of the WOMAC score [38, 44, 46, 68, 75, 79, 87]. The pooled data revealed that CHM plus NSAIDS was significantly more effective at reducing WOMAC than NSAIDS alone (SMD = −2.131; 95%CI = −3.082 to − 1.180; *P* ≤ 0.001; heterogeneity *χ*^2^ = 150.92, df = 6, *I*^2^ = 96.0%, *P* ≤ 0.001, Figure 4(a)). There were three studies comparing CHM and NSAIDS in terms of the WOMAC [61, 73, 83]. The pooled data showed that CHM monotherapy significantly decreased WOMAC when compared to NSAIDs alone (SMD = −0.672; 95%CI = −1.226 to − 0.119; *P* = 0.017; heterogeneity *χ*^2^ = 8.19, df = 2, *I*^2^ = 75.6%, *P* = 0.017, Figure 4(b)). Six studies compared CHM plus Glu with Glu alone in terms of WOMAC [33, 40, 47, 49, 59, 72]. The findings indicated that CHM plus Glu significantly decreased WOMAC score when compared to Glu alone (SMD = −1.315; 95%CI = −2.162 to − 0.468; *P* = 0.002; heterogeneity *χ*^2^ = 112.54, df = 5, *I*^2^ = 95.6%, *P* ≤ 0.001, Figure 4(c)). Six studies examined the efficacy of CHM and Glu in terms of WOMAC. The pooled data indicated that CHM monotherapy was significantly more effective than Glu in reducing WOMAC (SMD = −1.095; 95%CI = −1.607 to − 0.583; *P* ≤ 0.001; heterogeneity *χ*^2^ = 36.84, df = 5, *I*^2^ = 86.4%, *P* ≤ 0.001, Figure 4(d)). We used metaregression to determine the sources of heterogeneity in the findings. We also conducted metaregression to examine the year of publication, duration of treatment, and the sample size to ascertain the potential reasons of interstudy heterogeneity (Figure 5). Altogether, the year of publication (*β* = −0.112; *P* = 0.171; *R*^2^ = 4.83%), duration of treatment (*β* = 0.007; *P* = 0.861; *R*^2^ = 5.18%), and the sample size (*β* = −0.012; *P* = 0.303; *R*^2^ = 0.24%) were not significant predictors of heterogeneity for WOMAC.

#### 3.3.3. Lysholm Score

**Figure 6:**
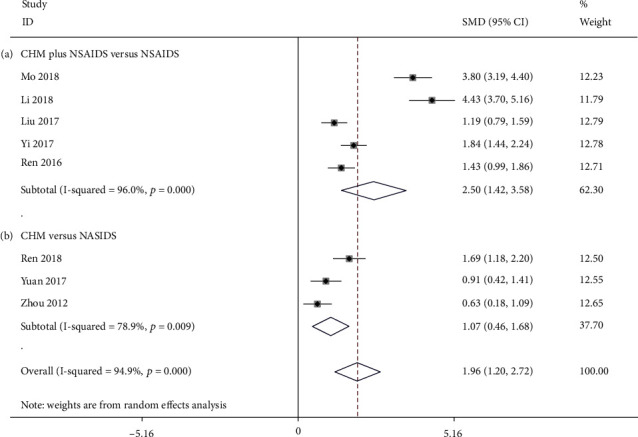
Forest plot of Lysholm score: (a) CHM plus NSAIDS versus NSAIDS and (b) CHM versus NSAIDS.

Five studies compared the Lysholm score between CHM plus NSAIDS and NSAIDS alone [44, 46, 53, 57, 75]. The pooled data indicated that CHM plus NSAIDS was significantly more effective at improving Lysholm score than NSAIDS alone (SMD = 2.503; 95%CI = 1.424 to 3.583; *P* ≤ 0.001; heterogeneity *χ*^2^ = 99.72, df = 4, *I*^2^ = 96.0%, *P* ≤ 0.001, Figure 6(a)). There were three studies comparing the Lysholm score of CHM to that of NSAIDS [56, 77, 85]. The pooled data revealed that CHM monotherapy significantly increased the Lysholm score much more than NSAIDS alone (SMD = 1.071; 95%CI = 0.459 to 1.683; *P* = 0.001; heterogeneity *χ*^2^ = 9.47, df = 2, *I*^2^ = 78.9%, *P* = 0.009, Figure 6(b)).

#### 3.3.4. Lequesne Index

**Figure 7:**
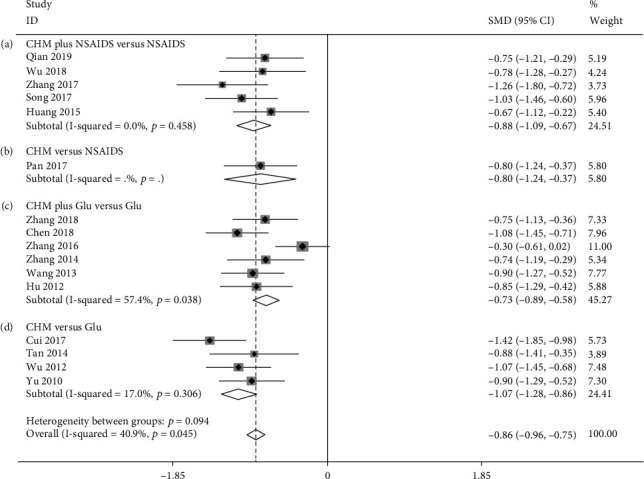
Forest plot of Lequesne index: (a) CHM plus NSAIDS versus NSAIDS, (b) CHM versus NSAIDS, (c) CHM plus Glu versus Glu, and (d) CHM versus Glu.

Five studies compared the effect of CHM plus NSAIDS to that of NSAIDS alone on the Lequesne index [42, 55, 60, 68, 81]. The pooled data revealed that CHM plus NSAIDS treatment significantly decreased the Lequesne index when compared to NSAIDS alone (SMD = −0.883; 95%CI = −1.095 to − 0.672; *P* ≤ 0.001; heterogeneity *χ*^2^ = 3.63, df = 4, *I*^2^ = 0%, *P* = 0.458, Figure 7(a)). There was only one study comparing the Lequesne index between CHM and NSAIDS [54]. The results indicated that CHM monotherapy significantly decreased the Lequesne index when compared to NSAIDS alone (SMD = −0.804; 95%CI = −1.239 to − 0.369; *P* ≤ 0.001, no heterogeneity, Figure 7(b)). Six studies compared the Lequesne index of CHM plus Glu to Glu alone [33, 41, 64, 78, 80, 82]. The available data demonstrated that CHM plus Glu significantly reduced the Lequesne index when compared to Glu alone (SMD = −0.734; 95%CI = −0.890 to − 0.579; *P* ≤ 0.001; heterogeneity *χ*^2^ = 11.74, df = 5, *I*^2^ = 57.4%, *P* = 0.038, Figure 7(c)). Based on the Lequesne index, four studies compared CHM versus Glu [35, 62, 69, 76]. The pooled results indicated that CHM monotherapy significantly reduced the Lequesne index when compared to Glu alone (SMD = −1.071; 95%CI = −1.283 to − 0.859; *P* ≤ 0.001; heterogeneity *χ*^2^ = 3.61, df = 3, *I*^2^ = 17.0%, *P* = 0.306, Figure 7(d)).

#### 3.3.5. Effective Rate

**Figure 8:**
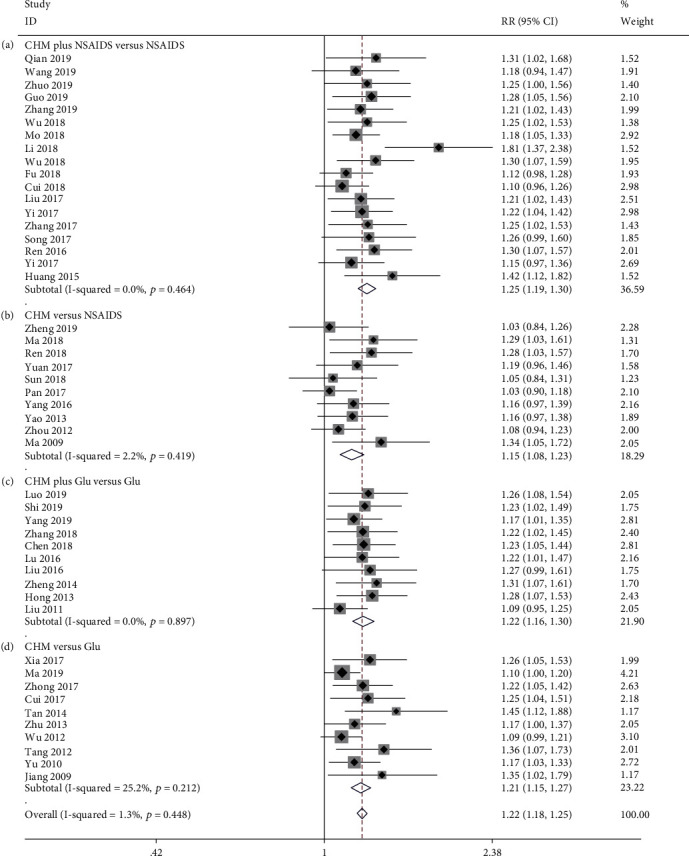
Forest plot of effective rate: (a) CHM plus NSAIDS versus NSAIDS, (b) CHM versus NSAIDS, (c) CHM plus Glu versus Glu, and (d) CHM versus Glu.

Eighteen studies examined the effectiveness of CHM plus NSAIDS to NSAIDS alone [34, 37, 38, 42, 44, 46, 53, 55, 57, 60, 65, 67, 68, 75, 79, 81, 87]. The pooled data demonstrated that CHM plus NSAIDS significantly increased the effective rate when compared to NSAIDS alone (RR = 1.247; 95%CI = 1.192 to 1.303; *P* ≤ 0.001; heterogeneity *χ*^2^ = 16.86, df = 17, *I*^2^ = 0%, *P* = 0.464, Figure 8(a)). There were ten studies comparing the effective rate between CHM and NSAIDS [50, 52, 54, 56, 61, 71, 73, 77, 83, 85]. The pooled data indicated that CHM monotherapy significantly improved the effective rate when compared to NSAIDS alone (RR = 1.154; 95%CI = 1.084 to 1.229; *P* ≤ 0.001; heterogeneity *χ*^2^ = 9.20, df = 9, *I*^2^ = 2.2%, *P* = 0.419, Figure 8(b)). Ten studies compared the effective rate between CHM plus Glu with Glu [33, 40, 45, 47–49, 59, 72, 80, 82]. The pooled data demonstrated that CHM plus Glu significantly increased the effective rate when compared to Glu alone (RR = 1.223; 95%CI = 1.156 to 1.295; *P* ≤ 0.001; heterogeneity *χ*^2^ = 4.21, df = 9, *I*^2^ = 0%, *P* = 0.897, Figure 8(c)). Ten studies compared the effective rate of CHM and Glu treatment [35, 43, 51, 62, 63, 69, 70, 76, 84, 86]. The pooled data demonstrated that CHM monotherapy significantly increased the effective rate when compared to Glu alone (RR = 1.208; 95%CI = 1.148 to 1.272; *P* ≤ 0.001; heterogeneity *χ*^2^ = 12.03, df = 9, *I*^2^ = 25.2%, *P* = 0.212, Figure 8(d)).

#### 3.3.6. Adverse Events

**Figure 9:**
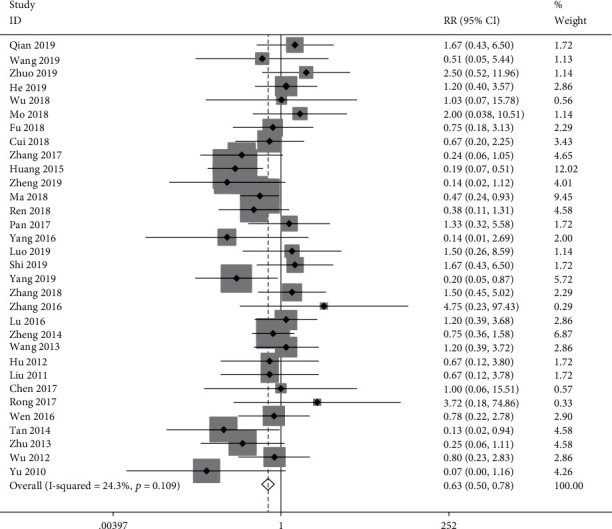
Forest plot of adverse events.

We identified adverse events in thirty-two studies. The trial group experienced 108/1489 adverse events, whereas the control group experienced 172/1472. The risk of adverse events was significantly lower in the CHM group than that in the control group (RR = 0.625; 95%CI = 0.500 to 0.783; *P* ≤ 0.001; heterogeneity *χ*^2^ = 40.94, df = 31, *I*^2^ = 24.3%, *P* = 0.109, Figure 9). Our findings indicated that the most often occurring adverse effects included gastrointestinal discomfort (nausea, diarrhea, thirst, poor appetite, stomach pain, and constipation), abnormal liver function, and rash. Significant adverse impacts that were mild, no severe adverse impacts, and death were reported in the included RCTs.

#### 3.3.7. Publication Bias and Sensitivity Analysis

**Figure 10:**
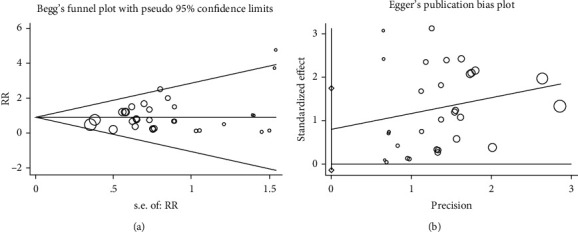
Begg's funnel plot (a) and Egger's test (b) of adverse events.

We examined the possibility of publication bias of the adverse effects in this meta-analysis using Begg's funnel plot and Egger's test (Figure 10). As a result of the symmetrical shape of the funnel plots and the *P* values from Begg's and Egger's tests, there was evidence of notable publication bias for adverse events (*P* = 0.661 and *P* = 0.847, respectively).

**Figure 11:**
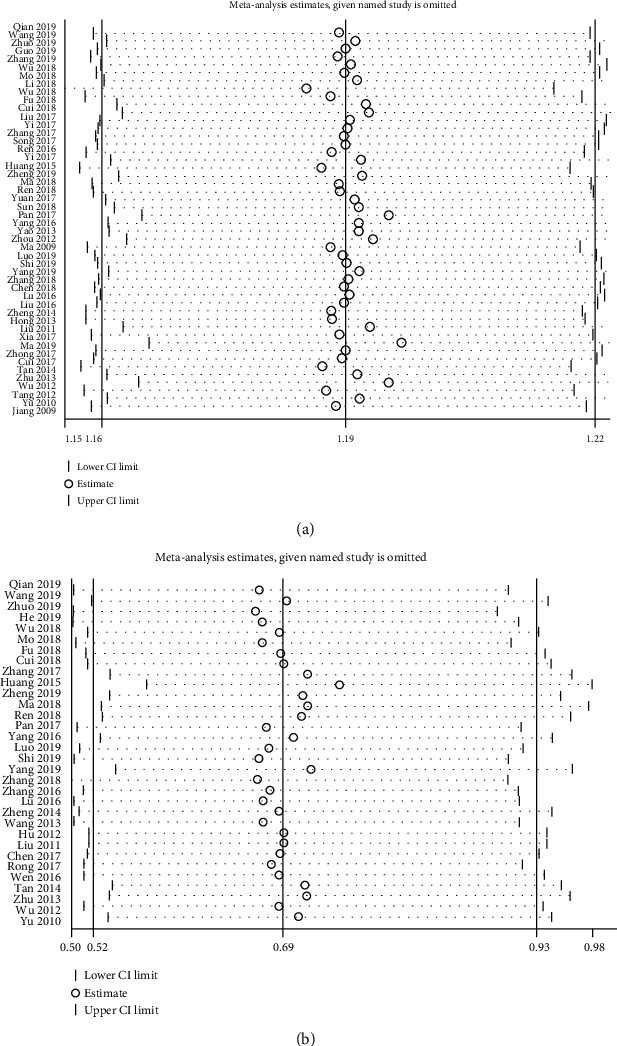
Sensitivity analysis for effective rate (a) and adverse events (b).

To establish the influence of each included study on the pooled RRs for the effective rate and adverse effects and to validate the robustness of our findings, we performed a sensitivity analysis by excluding one study at a time and computing the pooled RRs for the rest of the RCTs. The results of the sensitivity analysis indicated that excluding each study individually had no discernible influence on the pooled RRs showing that the findings of this meta-analysis are comparatively robust (Figure 11).

#### 3.3.8. Description of the CHMs

The most commonly used herbs across all formulae included Niu Xi (*Radix Achyranthis Bidentatae*, Twotooth *Achyranthes* root), Di Huang (*Radix Rehmanniae*, Rehmannia root), Dang Gui (*Radix Angelicae Sinensis*, Chinese angelica), Chuan Xiong (*Radix Ligustici Wallichii*, Sichuan lovage rhizome), Du Zhong (*Cortex Eucommiae Ulmoidis*, *Epimedium*), Gan Cao (*Radix Glycyrrhizae*, liquorice), Du Huo (*Radix Angelicae Pubescentis*, Pubescent angelica root), Sang Ji Sheng (*Radix Loranthi Seu visci*, Chinese taxillus twig), Bai Shao (*Radix Paeoniae Alba*, White paeony root), Fang Feng (*Radix Ledebouriellae Divaricatae*, Divaricate saposhnikovia root), Qin Jiao (*Radix Gentianae Macrophyllae*, Largeleaf gentian root), Fu Ling (*Poria Cocos Wolff*, Tuckahoe), Xi Xin (*Asari Radix et Rhizoma*, *Asarum*), Yin Yang Huo (*Epimedium brevicornu Maxim*, Icariin), Huang Qi (*Radix Astragali Membranacei*, *Astragalus*), Wei Ling Xian (*Radix Clematidis Chinensis*, Chinese clematis root), and Bu Gu Zhi (*Psoralea corylifolia Linn*, *Fructus psoraleae*) (Table 4).

## 4. Discussion

### 4.1. Summary of Evidence

Herein, we updated a systematic review and meta-analysis on the efficacy and safety of CHM treatment in patients with KOA. A total of fifty-six high-quality RCTs, including 5350 patients with KOA, were included in the analysis. Our primary findings indicated that using CHM as adjuvant therapy or monotherapy for KOA treatment reduced the VAS, WOMAC, and Lequesne index while improving the Lysholm score and overall effective rate. Additionally, we discovered that CHM adjuvant or monotherapy had fewer adverse effects than the controls, indicating that CHM was safe and effective in treating KOA. Therefore, we provide supporting evidence that, to a significant extent, CHM can potentially be recommended for use in KOA patients.

### 4.2. Comparison with Previous Studies

Significant research demonstrates that the oral and topical use of CHM is both safe and effective in the treatment of KOA. A meta-analysis of 23 RCTs including 2362 patients demonstrated that CHM is both safe and effective in alleviating pain, restoring function, and promoting health in patients with KOA [28]. Another systematic review found that Duhuo Jisheng decoction (DJD) combined with Western medicine or sodium hyaluronate injection was effective in treating KOA [88]. However, the effectiveness and safety of DJD remain debatable due to a scarcity of clinical trials and a lack of methodological rigor. Additionally, a Cochrane review of two RCTs including 327 patients found that orally bioavailable avocado soybean unsaponifiables (ASU) significantly relieved the pain symptoms in hip-OA patients when compared to a placebo. Additionally, this review established that the use of ASU helped patients in reducing their use of NSAIDs [89]. However, a primary concern in these earlier investigations has been the limited sample size and low quality. Therefore, in the current systematic review, we included 56 high-quality RCTs involving 5350 patients with KOA, which provides strong evidence that CHM is safe and effective for patients with KOA, consistent with the previous studies.

### 4.3. Strengths

The strengths of this meta-analysis study included a clearly defined research question, which minimized the bias in the selection of RCTs and improved the fidelity and consistency due to a precise research approach that we designed before the meta-analysis, an in-depth search of the literature, agreement between the two researchers on the entry data components, and quality control appraisal of all data. All of the studies included were RCTs with a significant proportion being of high quality. This assisted in overcoming the drawbacks associated with recall or selection bias in nonrandomized studies. Additionally, the total number of trials and the overall sample size were comparatively large (56 trials with 5350 patients). To ascertain the source of heterogeneity, we performed subgroup and metaregression analyses. Consequently, we found no evidence of publication bias in this meta-analysis, and sensitivity analysis revealed that the findings of this meta-analysis are comparatively robust.

### 4.4. Limitations

This study has several limitations. First, although RCTs were included, the primary studies included had certain inherent and methodological limitations; specifically, only 42 of the trials supplied sufficient information on the randomization process. The remaining RCTs, on the other hand, reported the allocation concealment. KOA is a chronic condition requiring lifelong treatment. Long-term efficacy and safety studies are critical for determining a drug's therapeutic usefulness. However, the duration of therapy, in this case, was between two and twelve weeks. Therefore, we were unable to assess the long-term safety of CHM for treating KOA since the duration of treatment in the included studies was short, and no dropouts were revealed in a significant percentage of the included studies. Thirdly, the formula composition, dosage, administration approaches, and duration of CHM treatments varied significantly in the primary RCTs. This clinical heterogeneity has the potential to jeopardize the validity of our findings. Fourthly, a significant proportion of the included RCTs did not involve a formal pretrial sample size calculation. Inadequate sample size in RCTs appears to be one risk factor for overestimating intervention benefits. Finally, we limited our search to studies published in English or Chinese repositories; therefore, studies published in other languages may have been overlooked. Additionally, because all RCTs included in the study were conducted in China, our findings may not be generalizable. Therefore, more multicenter RCTs of CHM for treating KOA are required to allow for global data generalization.

### 4.5. Implications for Practice

The evidence presented here indicates that using CHM as monotherapy or adjuvant treatment is beneficial and typically safe for treating KOA patients. We identified *Radix Achyranthis Bidentatae*, *Radix Rehmanniae*, *Radix Angelicae Sinensis*, *Radix Ligustici Wallichii*, *Cortex Eucommiae Ulmoidis*, *Radix Glycyrrhizae*, *Radix Angelicae Pubescentis*, *Radix Loranthi Seu visci*, *Radix Paeoniae Alba*, *Radix Ledebouriellae Divaricatae*, *Radix Gentianae Macrophyllae*, *Poria Cocos Wolff*, *Asari Radix et Rhizoma*, *Epimedium brevicornu Maxim*, *Radix Astragali Membranacei*, *Radix Clematidis Chinensis*, and *Psoralea corylifolia Linn* as the most frequently used herbs in KOA prescriptions., which should further be considered in the formulation of Chinese herbal prescriptions for KOA. Therefore, based on the high frequency of use of CHM for KOA, their therapeutic principles can guide CHM treatment for KOA, thereby increasing its effectiveness and safety.

### 4.6. Implications for Research

Here, we provide key concepts that are likely to stimulate further research in this field. Initiatives to increase the methodological quality of RCTs are urgently needed. We urge that in the future, recommendations such as the CONSORT Extension for Chinese Herbal Medicine Formulas 2017 [90], the CONSORT 2010 statement [91], and the protocols for designing RCTs to investigate CHM [92] be used to establish and report RCTs on CHM. Despite the finding that CHM therapy was reasonably safe for patients with KOA in the evaluated studies, further research is needed to corroborate the safety of CHM for KOA. Bian et al. [93] established a standard format for reporting adverse drug reactions (ADR) in CHM, which is likely to enhance ADR reporting. Clinical trials and studies with a longer follow-up time are recommended to provide a complete understanding of the long-term safety profile of CHM in patients with KOA. Recent advances in integrative medicine have enabled research to be conducted on disease-syndrome combinations. The effectiveness of TCM practice is contingent upon accurate syndrome differentiation. Therefore, an excellent distinction of disease symptoms is required for drug prescriptions [94]. Accurate syndrome differentiation of KOA should be performed during the evaluation of the safety and efficacy of CHM treatment. Individualized TCM prescriptions will give satisfactory treatment for specific diseases. For example, a study by Bensoussan et al. [95] published in JAMA showed that using personalized CHM to treat irritable bowel syndrome was superior to common hypnotic prescriptions. Thus, in future clinic practice, a suitable selection of medications among the 17 most often used herbs is recommended based on syndrome-specific characteristics. This will improve the efficacy of CHM in the treatment of KOA.

## 5. Conclusion

Our systematic and meta-analysis study offers supportive evidence that CHM, either adjuvant therapy or monotherapy, reduces the VAS, WOMAC, and Lequesne index and improves the Lysholm score and overall effective rate in patients with KOA. Additionally, CHM was well tolerated and safe in KOA patients. We found frequently used CHMs that might contribute to the formulation of a herbal formula that could be considered for further clinical use. However, given the heterogeneity and limited sample size in this study, larger multicenter and high-quality RCTs are needed to validate the benefits of CHM in the treatment of KOA.

## Figures and Tables

**Table 1:** The characteristics of the included studies.

Study	Study design	Diagnostic criteria	Sample size	Sample and characteristics (male/female; mean age, year)		Interventions		Disease duration	Course of treatment	Outcome index
				TG	CG	TG	CG			
Chen 2017 [28]	RCT	CRA criteria (2003)	92	46 (16/30); 51.2 ± 6.5	46 (20/26); 53.1 ± 7.2	Sanbi Xiao granule (0.3 g, tid, 4 w)	Glucosamine sulfate (628 mg, tid, 4 w)	TG: 6.3 ± 3.4 y CG: 7.1 ± 3.9 y	4 w	(1) VAS (2) WOMAC (3) ADR
Chen 2018 [29]	RCT	COA criteria (2007)	128	64 (26/38); 56.1	64 (24/40); 57.3	(1) Danqi granule (12 g, bid, 24 w) (2) Glucosamine sulfate (500 mg, tid, 24 w)	Glucosamine sulfate (500 mg, tid, 24 w)	TG: 0.6 to 9 y CG: 0.7 to 8 y	24 w	(1) WOMAC (2) ER (3) VAS (4) Lequesne
Cui 2017 [31]	RCT	ACR criteria (1986)	106	66 (30/36); 61.2 ± 8.60	40 (17/23); 55.32 ± 8.65	Chaihu Jiangu decoction (1 package, qod, 12 w)	Glucosamine hydrochloride (750 mg, bid, 12 w)	TG: 10.6 y CG: 9.9 y	12 w	(1) VAS (2) ER (3) Lequesne
Cui 2018 [30]	RCT	COA criteria (2007)	122	61 (30/31); 64.7 ± 6.92	61 (32/29); 62.5 ± 8.16	(1) Bushen Huoxue decoction (1 package, bid, 12 w) (2) Diclofenac sodium (75 mg, qd, 12 w)	Diclofenac sodium (75 mg, qd, 12 w)	TG: 3.12 ± 1.28 y CG: 2.83 ± 1.69 y	12 w	(1) ER (2) ADR
Dou 2015 [32]	RCT	COA criteria (2007)	60	30 (14/16); 64	30 (17/13); 63	Danzi Kangxi electuary (6 g, tid, 4 w)	Glucosamine sulfate (500 mg, tid, 4 w)	TG: 2 to 6 y CG: 3 to 5 y	4 w	(1) VAS (2) ER
Fu 2018 [33]	RCT	ACR criteria (1995)	76	38 (13/25); 69 ± 4.96	38 (14/24); 70 ± 4.13	(1) Duhuo Jisheng decoction (1 package, bid, 4 w) (2) Celecoxib (200 mg, qd, 4 w)	Celecoxib (200 mg, qd, 4 w)	TG: 3.1 ± 1.5 y CG: 2.8 ± 1.9 y	4 w	(1) ER (2) ADR
Guo 2019 [34]	RCT	COA criteria (2007)	102	51 (31/20); 58.16 ± 5.24	51 (29/22); 57.94 ± 5.13	(1) Duhuo Jisheng decoction (1 package, bid, 4 w) (2) Celecoxib (200 mg, bid, 4 w)	Celecoxib (200 mg, bid, 4 w)	TG: 1.89 ± 0.74 y CG: 2.01 ± 0.76 y	4 w	(1) VAS (2) WOMAC (3) ER
He 2019 [35]	RCT	ACR criteria (1995)	70	35 (11/24); 52.2 ± 8.5	35 (12/23); 54.4 ± 7.9	(1) Shuanggu Sanzi capsule (0.9 g, tid, 4 w) (2) Celecoxib (200 mg, bid, 4 w)	Celecoxib (200 mg, bid, 4 w)	TG: 5.9 ± 1.1 y CG: 5.7 ± 1.4 y	4 w	(1) ADR
Hong 2013 [36]	RCT	ACR criteria (1995)	115	63 (25/38); 51.75 ± 7.84	52 (20/32); 52.54 ± 8.23	(1) Qufeng Jiangu decoction (1 package, qd, 12 w) (2) Glucosamine hydrochloride (480 mg, tid, 12 w)	Glucosamine hydrochloride (480 mg, tid, 12 w)	TG: 58.1 ± 34.7 m CG: 56.2 ± 33.0 m	12 w	(1) WOMAC (2) ER
Hu 2012 [37]	RCT	ACR criteria (1995)	90	45 (13/32); 58.73 ± 9.19	45 (16/29); 60.42 ± 9.21	(1) Bushen Zhuangu decoction (1 package, qd, 6 w) (2) Glucosamine hydrochloride (750 mg, bid, 6 w)	Glucosamine hydrochloride (750 mg, bid, 6 w)	NR	6 w	(1) Lequesne (2) VAS (3) ADR
Huang 2015 [38]	RCT	ACR criteria (1986)	80	40(NR); 58.8 ± 4.4	40(NR); 58.9 ± 4.3	(1) Bushen Guangjie Huoluo Pulvis (1 package, bid, 4 w) (2) Celecoxib (200 mg, bid, 4 w)	Celecoxib (200 mg, bid, 4 w)	TG: 4.5 ± 2.2 y CG: 4.6 ± 2.1 y	4 w	(1) Lequesne (2) ER (3) ADR
Jiang 2009 [39]	RCT	ACR criteria (1995)	60	30 (11/19); 52.1 ± 7.1	30 (9/22); 53.6 ± 8.2	Bushen Huoxue decoction (1 package, qd, 24 w)	Glucosamine sulfate (628 mg, tid, 24 w)	TG: 4.16 ± 2.11y CG: 3.89 ± 2.56 y	24 w	(1) VAS (2) WOMAC (3) Lequesne (4) ER
Li 2018 [40]	RCT	COA criteria (2007)	100	50 (19/31); 59.72 ± 8.15	50 (23/27); 61.65 ± 7.43	(1) Duhuo Jisheng decoction (1 package, bid, 8 w) (2) Celecoxib (200 mg, qd, 8 w)	Celecoxib (200 mg, qd, 8 w)	TG: 40.34 ± 21.02 m CG: 39.43 ± 20.67 m	4 w	(1) WOMAC (2) ER (3) Lysholm
Liu 2011 [43]	RCT	ACR criteria (1995)	80	40 (19/21); 63 ± 8	40 (22/18); 62 ± 8	(1) Zhengqing Fengtongning tablet (60 mg, tid, 12 w) (2) Glucosamine hydrochloride (480 mg, tid, 12 w)	Glucosamine hydrochloride (480 mg, tid, 12 w)	TG: 32 ± 6 m CG: 31 ± 5 m	12 w	(1) WOMAC (2) ER (3) ADR
Liu 2016 [41]	RCT	COA criteria (2007)	90	45 (25/20); 55.22 ± 3.95	45 (21/24); 54.29 ± 3.56	(1) Jingu Tongning capsule (0.5 g, tid, 12 w) (2) Glucosamine sulfate (628 mg, tid, 12 w)	Glucosamine sulfate (628 mg, tid, 12 w)	TG: 3.93 ± 1.89 y CG: 3.89 ± 1.63 y	12 w	(1) VAS (2) ER (3) Lysholm
Liu 2017 [42]	RCT	CRA criteria (2010)	114	57 (35/22); 60.4 ± 5.2	57 (36/21); 61.2 ± 4.8	(1) Shujin decoction (1 package, qd, 4 w) (2) Celecoxib (200 mg, qd, 4 w)	Celecoxib (200 mg, qd, 4 w)	TG: 5.6 ± 1.7 y CG: 5.2 ± 1.4 y	4 w	(1) VAS (2) WOMAC (3) Lysholm (4) ER
Lu 2016 [44]	RCT	CRA criteria (2010)	100	50 (33/17); 64.53 ± 6.84	50 (35/15); 65.78 ± 5.93	(1) Lujiao Zhuanggu capsule (1.5 g, tid, 8 w) (2) Glucosamine hydrochloride (480 mg, tid, 8 w)	Glucosamine hydrochloride (480 mg, tid, 8 w)	TG: 9.86 ± 1.75 m CG: 9.76 ± 1.63 m	8 w	(1) VAS (2) ER (3) ADR
Luo 2019 [45]	RCT	COA criteria (2007)	98	49 (13/36); 57.49 ± 10.52	49 (14/35); 59.92 ± 10.89	(1) Zhengqing Fengtongning tablet (60 mg, bid, 12 w) (2) Glucosamine hydrochloride (750 mg, tid, 12 w)	Glucosamine hydrochloride (750 mg, tid, 12 w)	TG: 4.79 ± 0.88 y CG: 4.99 ± 0.91 y	12 w	(1) WOMAC (2) ER (3) ADR
Ma 2009 [48]	RCT	CRA criteria (2003)	118	59 (5/54); 52.07 ± 17.29	59 (7/52); 51.34 ± 18.46	Huoxue Tongluo decoction (1 package, qd, 12 w)	Celecoxib (200 mg, qd, 12 w)	TG: 7.03 ± 3.23 y CG: 7.21 ± 3.13 y	12 w	(1) VAS (2) ER
Ma 2018 [46]	RCT	COA criteria (2007)	60	32 (22/10); 53.29 ± 3.11	28 (19/9); 54.33 ± 3.46	Duhuo Jisheng decoction (1 package, qd, 12 w)	Celecoxib (200 mg, bid, 12 w)	TG: 50.87 ± 13.22 m CG: 51.29 ± 13.19 m	12 w	(1) VAS (2) ER (3) ADR
Ma 2019 [47]	RCT	COA criteria (2007)	164	82 (38/44); 67.0 ± 4.0	82 (40/42); 67.4 ± 3.8	Bushen Yiqi Huayu Jiedu decoction (1 package, qd, 8 w)	Glucosamine hydrochloride (480 mg, tid, 8 w)	TG: 7.4 ± 2.9 y CG: 7.2 ± 2.8 y	8 w	(1) VAS (2) Lysholm (3) ER
Mo 2018 [49]	RCT	COA criteria (2007)	120	60 (26/34); 58.94 ± 12.1	60 (25/35); 58.4 ± 11.6	(1) Kangguzhi Zengsheng capsule (17.5 g, tid, 12 w) (2) Celecoxib (200 mg, bid, 12 w)	Celecoxib (200 mg, bid, 12 w)	TG: 4.3 ± 1.6 w CG: 4.1 ± 1.4 w	12 w	(1) VAS (2) ER (3) ADR (4) Lysholm
Pan 2017 [22]	RCT	CRA criteria (2010)	80	40 (6/34); 64.53 ± 6.47	40 (5/35); 64.55 ± 5.57	Longbie capsule (1.2 g, tid, 4 w)	Celecoxib (200 mg, qd, 4 w)	TG: 14.53 ± 5.88 m CG: 15.30 ± 6.03 m	4 w	(1) VAS (2) Lequesne (3) ER (4) ADR
Qian 2019 [51]	RCT	COA criteria (2007)	78	39 (17/22); 49.12 ± 1.23	39 (19/20); 48.88 ± 1.19	(1) Yishen Quyu decoction (1 package, bid, 2 w) (2) Celecoxib (200 mg, qd, 2 w)	Celecoxib (200 mg, qd, 2 w)	TG: 2.12 ± 0.12 y CG: 2.11 ± 0.13 y	2 w	(1) VAS (2) Lequesne (3) ER (4) ADR
Ren 2016 [53]	RCT	CRA criteria (2005)	95	48 (34/14); 58.19 ± 7.16	47 (30/17); 57.34 ± 6.42	(1) Yishen Quyu decoction (1 package, bid, 4 w) (2) Celecoxib (200 mg, bid, 4 w)	Celecoxib (200 mg, bid, 4 w)	TG: 3.68 ± 1.19 y CG: 3.83 ± 1.13 y	4 w	(1) Lysholm (2) ER
Ren 2018 [52]	RCT	COA criteria (2007)	80	40 (18/22); 47.9 ± 2.2	40 (20/20); 48.1 ± 2.3	Duhuo Jisheng decoction (1 package, qd, 5 w)	Celecoxib (200 mg, qd, 5 w)	TG: 19.7 ± 3.2 m CG: 19.5 ± 3.4 m	5 w	(1) VAS (2) Lysholm (3) ER (4) ADR
Rong 2017 [54]	RCT	CRA criteria (2010)	73	42 (19/23); 53.68 ± 8.49	31 (14/17); 53.19 ± 6.78	Duhuo Jisheng decoction (1 package, qd, 4 w)	Glucosamine sulfate (500 mg, tid, 4 w)	NR	4 w	(1) VAS (2) WOMAC (3) ADR
Shi 2019 [55]	RCT	CRA criteria (2010)	78	39 (22/17); 59.79 ± 4.54	39 (24/15); 59.74 ± 4.63	(1) Duhuo Jisheng decoction (1 package, qd, 4 w) (2) Glucosamine sulfate (500 mg, tid, 4 w)	Glucosamine sulfate (500 mg, tid, 4 w)	TG: 2.73 ± 1.02 y CG: 2.62 ± 0.98 y	4 w	(1) WOMAC (2) ER (3) ADR
Song 2017 [56]	RCT	COA criteria (2007)	95	50 (28/22); 52.1 ± 3.8	45 (27/18); 52.5 ± 3.7	(1) Duhuo Jisheng decoction (1 package, qd, 4 w) (2) Celecoxib (200 mg, qd, 4 w)	Celecoxib (200 mg, qd, 4 w)	TG: 1.2 ± 0.5 y CG: 1.2 ± 0.6 y	4 w	(1) VAS (2) Lequesne (3) ER
Sun 2018 [57]	RCT	COA criteria (2007)	50	25 (12/13); 54.46 ± 8.25	25 (11/14); 55.37 ± 8.47	Qinbi decoction (1 package, qd, 4 w)	Celecoxib (200 mg, qd, 4 w)	TG: 9.40 ± 1.27 y CG: 8.93 ± 2.02 y	4 w	(1) VAS (2) WOMAC (3) ER
Tan 2014 [58]	RCT	ACR criteria (1995)	60	30 (8/22); 55.6	30 (12/18); 57.4	Huangqi Biejia pill (15 g, tid, 6 w)	Glucosamine hydrochloride (480 mg, tid, 6 w)	TG: 2.51 y CG: 2.42 y	6 w	(1) Lequesne (2) ER (3) ADR
Tang 2012 [59]	RCT	ACR criteria (1995)	114	56 (20/36); 57.2 ± 5.43	58 (22/36); 59.3 ± 4.34	Jiedu Yishen pill (15 g, bid, 8 w)	Glucosamine sulfate (500 mg, tid, 8 w)	TG: 5.1 ± 3.2 y CG: 4.5 ± 3.9 y	8 w	(1) WOMAC (2) ER
Wang 2013 [60]	RCT	ACR criteria (1986)	120	60 (35/25); 53.3 ± 5.1	60 (28/32); 54.5 ± 6.2	(1) Fufang Xiatian Wu pill (0.6 g, tid, 24 w) (2) Glucosamine hydrochloride (480 mg, bid, 24 w)	Glucosamine hydrochloride (480 mg, bid, 24 w)	TG: 5.71 ± 2.19 y CG: 5.88 ± 2.63 y	24 w	(1) Lequesne (2) ADR
Wang 2019 [61]	RCT	COA criteria (2007)	96	48 (10/38); 56.9	48 (13/35); 58.4	(1) Taoren Xikang pill (6 g, bid, 12 w) (2) Celecoxib (200 mg, bid, 4 w)	Celecoxib (200 mg, bid, 4 w)	NR	12 w	(1) VAS (2) WOMAC (3) ER (4) ADR
Wen 2016 [62]	RCT	ACR criteria (1995)	142	72 (29/43); 48.7 ± 12.1	70 (26/44); 49.2 ± 11.9	Duhuo Jisheng decoction (1 package, qd, 4 w)	Glucosamine hydrochloride (480 mg, tid, 4 w)	TG: 73.0 ± 18.5 d CG: 71.2 ± 16.8 d	4 w	(1) VAS (2) ER (3) ADR
Wu 2012 [65]	RCT	ACR criteria (1995)	120	60 (22/38); 61.2 ± 4.22	60 (19/41); 62.3 ± 6.51	Zhuanggu Tongbi pill (6 g, bid, 12 w)	Glucosamine sulfate (628 mg, tid, 12 w)	NR	12 w	(1) Lequesne (2) ER (3) ADR
Wu 2018 [63]	RCT	CRA criteria (2003)	93	47 (20/27); 58.93 ± 3.56	46 (18/28); 57.83 ± 7.15	(1) Bushen Huoxue decoction (1 package, bid, 12 w) (2) Celecoxib (200 mg, qd, 12 w)	Celecoxib (200 mg, qd, 12 w)	TG: 7.69 ± 1.82 y CG: 6.87 ± 1.45 y	12 w	(1) WOMAC (2) ER
Wu 2018 [64]	RCT	COA criteria (2007)	64	32 (12/20); 57.36 ± 4.89	32 (11/21); 57.98 ± 4.95	(1) Sanqi Xuejie capsule (4 capsules, tid, 4 w) (2) Celecoxib (200 mg, bid, 4 w)	Celecoxib (200 mg, bid, 4 w)	TG: 3.56 ± 0.74 y CG: 3.65 ± 0.78 y	4 w	(1) Lequesne (2) ER (3) ADR
Xia 2017 [66]	RCT	COA criteria (2007)	92	46 (20/26); 46.02 ± 11.81	46 (21/25); 45.96 ± 11.62	(1) Jingu Tongning capsule (1.5 g, tid, 4 w) (2) Glucosamine hydrochloride (480 mg, tid, 4 w)	Glucosamine hydrochloride (480 mg, tid, 4 w)	TG: 19.46 ± 4.89 m CG: 19.53 ± 4.82 m	4 w	(1) WOMAC (2) ER
Yang 2016 [67]	RCT	COA criteria (2007)	96	48 (15/33); 55.4 ± 9.8	48 (13/35); 56.6 ± 10.5	Jiawei Simiao Pulvis (1 package, bid, 4 w)	Celecoxib (200 mg, bid, 4 w)	TG: 5.6 ± 3.8 y CG: 6.5 ± 4.1 y	4 w	(1) VAS (2) ER (3) ADR
Yang 2019 [68]	RCT	COA criteria (2007)	120	60 (32/28); 51.06 ± 9.01	60 (27/33); 50.04 ± 11.04	(1) Duhuo Jisheng decoction (1 package, bid, 4 w) (2) Glucosamine sulfate (628 mg, tid, 4 w)	Glucosamine sulfate (628 mg, tid, 4 w)	TG: 6.1 ± 5.3 y CG: 6.1 ± 4.2 y	4 w	(1) WOMAC (2) ER (3) ADR (4) VAS
Yao 2013 [69]	RCT	COA criteria (2007)	81	41 (NR); NR	40 (NR); NR	Duhuo Jisheng decoction (1 package, qd, 6 w)	Celecoxib (200 mg, qd, 6 w)	NR	6 w	(1) VAS (2) WOMAC (3) ER
Yi 2017 [70]	RCT	ACR criteria (1986)	120	60 (21/39); 58	60 (23/37); 56	(1) Bushen Huoxue decoction (1 package, qd, 4 w) (2) Celecoxib (200 mg, qd, 4 w)	Celecoxib (200 mg, qd, 4 w)	NR	4 w	(1) VAS (2) WOMAC (3) ER
Yi 2017 [71]	RCT	COA criteria (2007)	136	68 (38/30); 60.2 ± 3.1	68 (37/31); 59.4 ± 2.3	(1) Qiangjin Zhuanggu decoction I (1 package, qd, 4 w) (2) Celecoxib (200 mg, qd, 4 w)	Celecoxib (200 mg, qd, 4 w)	TG: 7.5 ± 1.2 y CG: 7.7 ± 1.3 y	4 w	(1) VAS (2) WOMAC (3) Lysholm (4) ER
Yu 2010 [72]	RCT	ACR criteria (1995)	113	56 (21/35); 56 ± 17	57 (18/38); 59 ± 18	Duhuo Jisheng decoction (1 package, qd, 4 w)	Glucosamine sulfate (500 mg, tid, 4 w)	TG: 11 ± 5 y CG: 12 ± 6 y	4 w	(1) Lequesne (2) ER (3) ADR
Yuan 2017 [73]	RCT	COA criteria (2007)	70	35 (18/17); 48.3 ± 5.6	35 (15/20); 41.2 ± 4.8	Bushen Huoxue decoction (1 package, qd, 112 w)	Celecoxib (200 mg, qd, 12 w)	TG: 1.8 ± 1.2 y CG: 1.7 ± 1.1 y	12 w	(1) VAS (2) Lysholm (3) ER
Zhang 2016 [74]	RCT	COA criteria (2007)	156	80 (30/50); 53.1	76 (24/52); 51.4	(1) Jinwu Gutong capsule (0.3 g, tid, 12 w) (2) Glucosamine hydrochloride (750 mg, bid, 12 w)	Glucosamine hydrochloride (750 mg, bid, 12 w)	NR	12 w	(1) ER (2) Lequesne
Zhang 2017 [77]	RCT	COA criteria (2007)	63	32 (19/13); 65.86 ± 7.74	31 (20/11); 65.70 ± 7.69	(1) Fugui Gutong capsule (1.32 g, tid, 4 w) (2) Celecoxib (200 mg, qd, 4 w)	Celecoxib (200 mg, qd, 4 w)	TG: 2.20 ± 0.74 y CG: 2.08 ± 0.91 y	4 w	(1) VAS (2) Lequesne (3) ER (4) ADR
Zhang 2018 [76]	RCT	COA criteria (2007)	110	55 (21/34); 55.2	55 (20/35); 54.5	(1) Qianggu capsule (0.25 g, tid, 12 w) (2) Glucosamine sulfate (500 mg, tid, 12 w)	Glucosamine sulfate (500 mg, tid, 12 w)	TG: 5.41 ± 1.31 y CG: 5.66 ± 1.22 y	12 w	(1) VAS (2) ER (3) ADR (4) Lequesne
Zhang 2019 [75]	RCT	ACR criteria (1995)	86	43 (17/26); 50.32 ± 6.65	43 (15/28); 49.68 ± 6.12	(1) Duhuo Jisheng decoction (1 package, tid, 8 w) (2) Meloxicam (7.5 mg, bid, 8 w)	Meloxicam(7.5 mg, bid, 8 w)	TG: 70.52 ± 10.68 d CG: 68.20 ± 11.03 d	8 w	(1) WOMAC (2) ER
Zheng 2014 [78]	RCT	ACR criteria (1986)	80	40 (11/29); 60.8 ± 6.6	40 (9/31); 61.2 ± 5.8	(1) Zhengqing Fengtongning tablet (60 mg, tid, 4 w)	Glucosamine sulfate (628 mg, tid, 4 w)	TG: 12.6 ± 2.6 y CG: 11.9 ± 3.7 y	4 w	(1) Lequesne (2) ER (3) ADR
Zheng 2019 [79]	RCT	COA criteria (2007)	100	50 (27/23); 63.26 ± 7.42	50 (24/26); 63.12 ± 4.19	Bushen Huoxue decoction (1 package, bid, 4 w)	Celecoxib (200 mg, bid, 4 w)	TG: 32.37 ± 19.46 m CG: 33.24 ± 20.17 m	4 w	(1) VAS (2) WOMAC (3) ER (4) ADR
Zhong 2017 [80]	RCT	ACR criteria (1995)	116	58 (26/32); 56.2 ± 8.3	58 (28/30); 55.6 ± 8.5	Shentong Zhuyu decoction (1 package, qd, 4 w)	Glucosamine hydrochloride (750 mg, tid, 4 w)	TG: 3.3 ± 1.8 y CG: 3.3 ± 1.5 y	4 w	(1) VAS (2) ER
Zhou 2012 [81]	RCT	ACR criteria (1986)	78	43 (17/26); 53.61 ± 6.37	35 (14/21); 54.18 ± 6.13	Shufu Jiangu decoction (1 package, qd, 8 w)	Celecoxib (200 mg, qd, 8 w)	TG: 4.38 ± 1.52 y CG: 4.24 ± 1.49 y	8 w	(1) VAS (2) Lysholm (3) ER
Zhu 2013 [82]	RCT	ACR criteria (1995)	86	43 (8/22); 65.17 ± 8.73	43 (12/18); 64.93 ± 9.12	Zhengqing Fengtongning tablet (60 mg, bid, 12 w)	Glucosamine hydrochloride (480 mg, tid, 12 w)	TG: 16.1 ± 3.73 m CG: 15.3 ± 3.86 m	12 w	(1) WOMAC (2) ER (3) ADR
Zhuo 2019 [83]	RCT	COA criteria (2007)	64	32 (13/19); 60.90 ± 3.05	32 (14/18); 61.06 ± 2.86	(1) Rendong Bixie decoction (1 package, bid, 4 w) (2) Celecoxib (200 mg, qd, 4 w)	Celecoxib (200 mg, qd, 4 w)	TG: 7.28 ± 2.45 y CG: 6.50 ± 1.68 y	4 w	(1) WOMAC (2) ER (3) ADR

RCT: randomized controlled trial; TG: trial group; CG: control group; COA: Chinese Orthopedic Association; CRA: Chinese Rheumatology Association; ACR: American College of Rheumatology; VAS: visual analog score; WOMAC: Western Ontario and McMaster Universities Osteoarthritis Index; ER: effective rate; ADR: adverse drug reaction; NR: not reported.

**Table 2:** Assessment of study quality and risk of bias.

Study	7-item criteria							
	A	B	C	D	E	F	G	Total
Chen 2017 [28]	?	–	?	+	+	+	+	4
Chen 2018 [29]	+	?	+	+	+	+	+	6
Cui 2017 [31]	+	?	+	?	+	+	+	5
Cui 2018 [30]	?	?	+	+	+	+	+	5
Dou 2015 [32]	+	+	+	+	+	+	+	7
Fu 2018 [33]	+	–	+	?	+	+	+	5
Guo 2019 [34]	+	+	–	–	+	+	+	5
He 2019 [35]	?	?	+	+	+	–	+	4
Hong 2013 [36]	?	?	+	+	+	+	+	5
Hu 2012 [37]	?	?	+	+	+	+	+	5
Huang 2015 [38]	?	–	+	–	+	+	+	4
Jiang 2009 [39]	?	?	+	+	+	+	?	4
Li 2018 [40]	?	–	–	+	+	+	+	4
Liu 2011 [43]	?	?	+	+	+	+	–	4
Liu 2016 [41]	?	?	+	+	+	+	+	5
Liu 2017 [42]	?	+	+	+	+	+	+	6
Lu 2016 [44]	?	?	?	+	+	+	+	4
Luo 2019 [45]	+	?	+	–	+	–	+	4
Ma 2009 [48]	?	?	+	+	?	+	+	4
Ma 2018 [46]	+	+	–	–	+	+	+	5
Ma 2019 [47]	+	?	+	+	+	+	+	6
Mo 2018 [49]	+	?	+	?	+	+	+	5
Pan 2017 [22]	+	–	+	–	+	+	+	5
Qian 2019 [51]	+	–	+	+	+	+	+	6
Ren 2016 [53]	?	+	+	+	+	–	+	5
Ren 2018 [52]	+	?	–	–	+	+	+	4
Rong 2017 [54]	?	+	–	–	+	+	+	4
Shi 2019 [55]	+	–	+	+	+	+	+	6
Song 2017 [56]	+	–	+	+	+	+	+	6
Sun 2018 [57]	+	–	+	+	+	+	+	6
Tan 2014 [58]	?	?	+	–	+	+	+	4
Tang 2012 [59]	+	?	+	?	+	+	+	5
Wang 2013 [60]	?	?	+	+	+	?	+	4
Wang 2019 [61]	+	–	?	+	+	+	+	5
Wen 2016 [62]	+	–	+	+	+	+	+	6
Wu 2012 [65]	+	+	+	+	+	+	+	7
Wu 2018 [63]	+	–	–	–	+	+	+	4
Wu 2018 [64]	+	?	+	?	+	+	+	5
Xia 2017 [66]	?	?	–	+	+	+	+	4
Yang 2016 [67]	?	?	–	+	+	+	+	4
Yang 2019 [68]	+	?	+	–	+	+	+	5
Yao 2013 [69]	+	?	+	+	+	+	+	6
Yi 2017 [70]	+	–	+	+	+	+	+	6
Yi 2017 [71]	?	?	+	–	+	+	+	4
Yu 2010 [72]	?	–	+	+	+	+	–	4
Yuan 2017 [73]	+	?	+	?	+	+	+	5
Zhang 2016 [74]	?	?	+	–	+	+	+	4
Zhang 2017 [77]	?	–	+	+	+	+	+	5
Zhang 2018 [76]	+	–	?	–	+	+	+	4
Zhang 2019 [75]	+	+	–	–	+	–	+	4
Zheng 2014 [78]	?	?	+	+	+	–	+	4
Zheng 2019 [79]	+	+	+	+	+	+	+	7
Zhong 2017 [80]	+	?	+	+	+	+	+	6
Zhou 2012 [81]	?	–	+	+	+	+	+	5
Zhu 2013 [82]	+	–	+	+	+	+	+	6
Zhuo 2019 [83]	?	–	?	+	+	+	+	4

A to G, the 7-item criteria. A: random sequence generation; B: allocation concealment; C: blinding of participants and personnel; D: blinding of outcome assessment; E: incomplete outcome data; F: selective reporting; G: other bias; +: low risk of bias, –: high risk of bias; ?: uncertain risk of bias.

**Table 3:** Ingredients of CHM in the included studies.

Study	Prescription name	Ingredients of herb prescription	Preparations	Quality control
Chen 2017 [28]	Sanbi Xiao granule	*Angelica sinensis*, *Radix Paeoniae Alba*, *Radix Rehmanniae Recen*, Ginseng, *Astragalus*, *Achyranthes* root, *Eucommia ulmoides*	Granule	Guangdong FDA approval number: ZB20060360
Chen 2018 [29]	Danqi granule	*Radix Rehmanniae Praeparata*, *Cornus officinalis*, *Rhizoma alismatis*, Chinese yam, *Epimedium*, *Paeonia suffruticosa*, Tuckahoe, Barbary wolfberry fruit, Semen Cuscutae, herba *Cistanche*, *Concha ostreae*	Granule	SFDA approval number: Z20050537
Cui 2017 [31]	Chaihu Jiangu decoction	*Radix Bupleuri*, *Scutellaria baicalensis*, *Rhizoma Pinellinae Praeparata*, *Codonopsis pilosula*, Cassia twig, *Radix Paeoniae Alba*, *Rhizoma Chuanxiong*, *Achyranthes* root, *Arisaema cum* bile, liquorice	Decoction	Hospital preparation
Cui 2018 [30]	Bushen Huoxue decoction	*Radix Rehmanniae Praeparata* 15 g, *Epimedium* 15 g, Semen Cuscutae 15 g, *Angelica sinensis* 10 g, *Salviae miltiorrhizae* 10 g, *Achyranthes* root 10 g, *Eucommia ulmoides* 10 g, *Radix Dipsaci* 10 g, *Radix Codonopsis* 15 g, *Rhizoma Atractylodis macrocephalae* 15 g, liquorice 6 g	Decoction	Hospital preparation
Dou 2015 [32]	Danzi Kangxi electuary	Human placenta, *Salviae miltiorrhizae*, *Radix Rehmanniae Praeparata*, *Achyranthes* root, *Fructus psoraleae*, *Morinda officinalis*, parasitic *Loranthus*, woodlouse, *Radix Angelicae Pubescentis*, *Radix Paeoniae Alba*	Electuary	Hospital preparation
Fu 2018 [33]	Duhuo Jisheng decoction	*Radix Angelicae Pubescentis* 15 g, parasitic *Loranthus* 15 g, glossy privet fruit 15 g, Semen Cuscutae 15 g, *Angelica sinensis* 15 g, *Eucommia ulmoides* 15 g, Barbary wolfberry fruit 15 g, *Radix Sileris* 15 g, Semen Psoraleae 15 g, *Achyranthes* root 15 g, peach seed 10 g, flowers carthami 10 g, *Rhizoma Chuanxiong* 10 g, herba Lycopi 10 g, liquorice 6 g, *Asarum* 3 g	Decoction	Hospital preparation
Guo 2019 [34]	Duhuo Jisheng decoction	*Radix Angelicae Pubescentis* 15 g, parasitic *Loranthus* 15 g, glossy privet fruit 15 g, Semen Cuscutae 15 g, *Angelica sinensis* 15 g, *Eucommia ulmoides* 15 g, Barbary wolfberry fruit 15 g, *Radix Sileris* 15 g, Semen Psoraleae 15 g, *Achyranthes* root 15 g, peach seed 10 g, flowers carthami 10 g, *Rhizoma Chuanxiong* 10 g, herba Lycopi 10 g, liquorice 6 g, *Asarum* 3 g	Decoction	Hospital preparation
He 2019 [35]	Shuanggu Sanzi capsule	Woodlouse, *Rhizoma Drynariae*, Pyritum, *Astragalus*, *Angelica sinensis*, Resina Draconis, *Rheum officinale*, olibanum, myrrh, *Rhizoma Cibotii*, Semen Allii Tuberosi, muskmelon seed, cucumber seed	Capsule	SFDA approval number: B20020788
Hong 2013 [36]	Qufeng Jiangu decoction	*Rhizoma seu Radix Notopterygii* 30 g, Caulis Spatholobi 30 g, *Radix Cynanchi Panicullati* 30 g, Rhizoma Chuanxiong 15 g, Rhizoma Curcumae 15 g, *Radix Curcumae* 15 g, Rhizoma Drynariae 15 g, obscured homalomena rhizome 15 g, Berba Aristolochiae Mollissimae 15 g, liquorice 6 g, Pericarpium Citri Reticulatae 6 g	Decoction	Hospital preparation
Hu 2012 [37]	Bushen Zhuangu decoction	*Radix Rehmanniae Praeparata* 15 g, *Epimedium* 15 g, Semen Cuscutae 15 g, *Angelica sinensis* 10 g, *Salviae miltiorrhizae* 10 g, *Achyranthes* root 10 g, *Eucommia ulmoides* 10 g, *Radix Dipsaci* 10 g, *Radix Codonopsis* 15 g, *Rhizoma Atractylodis macrocephalae* 15 g, liquorice 6 g	Decoction	Hospital preparation
Huang 2015 [38]	Bushen Guangjie Huoluo Pulvis	Semen Cuscutae 10 g, *Rhizoma Atractylodis macrocephalae* 20 g, *Morinda officinalis* 20 g, *Notoginseng Radix* 15 g, *Radix Curcumae Longae* 15 g, *Achyranthes* root 30 g, *Radix Arnebiae seu Lithospermi* 15 g, Rhizoma Alismatis 20 g, desert *Cistanche* 20 g, *Radix Sileris* 20 g, liquorice 5 g, Rhizoma Chuanxiong 20 g, *Radix Arnebiae seu Lithospermi* 15 g, cowherb seed 15 g	Pulvis	Hospital preparation
Jiang 2009 [39]	Bushen Huoxue decoction	*Radix Rehmanniae Praeparata* 15 g, *Epimedium* 15 g, Semen Cuscutae 15 g, *Angelica sinensis* 10 g, *Salviae miltiorrhizae* 10 g, *Achyranthes* root 10 g, *Eucommia ulmoides* 10 g, *Radix Dipsaci* 10 g, *Radix Codonopsis* 15 g, *Rhizoma Atractylodis macrocephalae* 15 g, liquorice 6 g	Decoction	Hospital preparation
Li 2018 [40]	Duhuo Jisheng decoction	*Radix Angelicae Pubescentis* 15 g, parasitic *Loranthus* 15 g, glossy privet fruit 15 g, Semen Cuscutae 15 g, *Angelica sinensis* 15 g, *Eucommia ulmoides* 15 g, Barbary wolfberry fruit 15 g, *Radix Sileris* 15 g, Semen Psoraleae 15 g, *Achyranthes* root 15 g, peach seed 10 g, flowers carthami 10 g, Rhizoma Chuanxiong 10 g, herba Lycopi 10 g, liquorice 6 g, *Asarum* 3 g	Decoction	Hospital preparation
Liu 2011 [43]	Zhengqing Fengtongning tablet	Sinomenine 60 mg	Tablet	SFDA approval number: Z20010174
Liu 2016 [41]	Jingu Tongning capsule	*Rheum officinale*, woodlouse, olibanum, myrrh, *Angelica sinensis*, flowers carthami, *Radix Paeoniae Alba*, *Fructus forsythiae*, *Fructus gardeniae*, borneol	Capsule	Henan FDA approval number: Z 204090031
Liu 2017 [42]	Shujin decoction	*Astragalus* 15 g, *Epimedium* 15 g, cornu *Cervi degelatinatum* 15 g, Rhizoma Chuanxiong 15 g, *Radix Rehmanniae Praeparata* 15 g, *Radix Angelicae Pubescentis* 15 g, *Radix Aconiti Lateralis Preparata* 10 g, *Lycopodium clavatum* 10 g, *Rhizoma seu Radix Notopterygii* 10 g, Barbary wolfberry fruit 10 g, *Angelica sinensis* 10 g, *Radix Sileris* 10 g, *Kadsura* pepper stem 8 g, *Radix Paeoniae Alba* 30 g, *Fructus cnidii* 12 g, Caulis Spatholobi 20 g, *Elecampane* 8 g, liquorice 8 g	Decoction	Hospital preparation
Lu 2016 [44]	Lujiao Zhuanggu capsule	Cornu Cervi Degelatinatum, *Fructus psoraleae*, *Astragalus*, wolfberry, Rhizoma Chuanxiong, *Rheum officinale*, *Radix Dipsaci*	Capsule	Guizhou EDA approval number: Z20120003
Luo 2019 [45]	Zhengqing Fengtongning tablet	Sinomenine 60 mg	Tablet	SFDA approval number: Z20010174
Ma 2009 [48]	Huoxue Tongluo decoction	Peach seed 10 g, flowers carthami 12 g, Rhizoma Chuanxiong 10 g, *Angelica sinensis* 12 g, root of common peony 10 g, *Achyranthes* root 12 g, parasitic *Loranthus* 15 g, *Eucommia ulmoides* 12g, *Radix Clematidis* 12 g	Decoction	Hospital preparation
Ma 2018 [46]	Duhuo Jisheng decoction	*Radix angelicae pubescentis* 15 g, Parasitic *loranthus* 15 g, glossy privet fruit 15 g, Semen cuscutae 15 g, *Angelica sinensis* 15 g, *Eucommia ulmoides* 15 g, Barbary Wolfberry fruit 15 g, *Radix Sileris* 15 g, semen psoraleae 15 g, *Achyranthes* root 15 g, peach seed 10 g, flowers carthami 10 g, Rhizoma Chuanxiong 10 g, Herba Lycopi 10 g, liquorice 6 g, *Asarum* 3 g	Decoction	Hospital preparation
Ma 2019 [47]	Bushen Yiqi Huayu Jiedu decoction	*Astragalus* 15 g, *Fructus psoraleae* 15 g, herba *Cistanche* 15 g, *Radix Codonopsis* 12 g, Rhizoma Chuanxiong 9 g, *Salviae miltiorrhizae* 9 g, synthetic musk 0.03 g	Decoction	Hospital preparation
Mo 2018 [49]	Kangguzhi Zengsheng capsule	*Radix Rehmanniae Praeparata*, desert Cistanche, Rhizoma Cibotii, glossy privet fruit, *Epimedium*, Caulis Spatholobi, Radish seed, Rhizoma Drynariae, *Achyranthes* root	Capsule	SFDA approval number: Z10980006
Pan 2017 [22]	Longbie capsule	Morinda officinalis, Rhizoma Curculiginis, Semen Cuscutae, Scorpio, centipede, *Agkistrodon*, woodlouse, *Salviae miltiorrhizae*, *Radix Aconiti Preparata*	Capsule	Guangdong FDA approval number: Z20071030
Qian 2019 [51]	Yishen Quyu decoction	Rhizoma Chuanxiong 18 g, Chinese angelica 18 g, root of *Achyranthes bidentata* 18 g, Rhizoma Drynariae 18 g, Rhizoma Cibotii 15 g, *Epimedium* 15 g, pubescent angelica root 12 g, *Eucommia ulmoides* 12 g, *Radix Dipsaci* 12 g	Decoction	Hospital preparation
Ren 2016 [53]	Yishen Quyu decoction	Rhizoma Chuanxiong 18 g, *Angelica sinensis* 18 g, *Achyranthes* root 18 g, Rhizoma Drynariae 18 g, Rhizoma Cibotii 15 g, *Epimedium* 15 g, *Radix Angelicae Pubescentis* 12 g, *Morinda officinalis* 12 g, *Eucommia ulmoides* 12 g, *Radix Dipsaci* 12 g	Decoction	Hospital preparation
Ren 2018 [52]	Duhuo Jisheng decoction	*Radix Angelicae Pubescentis* 15 g, parasitic *Loranthus* 15 g, glossy privet fruit 15 g, Semen Cuscutae 15 g, *Angelica sinensis* 15 g, *Eucommia ulmoides* 15 g, Barbary wolfberry fruit 15 g, *Radix Sileris* 15 g, Semen Psoraleae 15 g, *Achyranthes* root 15 g, peach seed 10 g, flowers carthami 10 g, Rhizoma Chuanxiong 10 g, herba Lycopi 10 g, liquorice 6 g, *Asarum* 3 g	Decoction	Hospital preparation
Rong 2017 [54]	Duhuo Jisheng decoction	*Radix Angelicae Pubescentis* 15 g, parasitic *Loranthus* 15 g, glossy privet fruit 15 g, Semen Cuscutae 15 g, *Angelica sinensis* 15 g, *Eucommia ulmoides* 15 g, Barbary wolfberry fruit 15 g, *Radix Sileris* 15 g, Semen Psoraleae 15 g, *Achyranthes* root 15 g, peach seed 10 g, flowers carthami 10 g, Rhizoma Chuanxiong 10 g, herba Lycopi 10 g, liquorice 6 g, *Asarum* 3 g	Decoction	Hospital preparation
Shi 2019 [55]	Duhuo Jisheng decoction	*Radix Angelicae Pubescentis* 15 g, parasitic *Loranthus* 15 g, glossy privet fruit 15 g, Semen Cuscutae 15 g, *Angelica sinensis* 15 g, *Eucommia ulmoides* 15 g, Barbary wolfberry fruit 15 g, *Radix Sileris* 15 g, Semen Psoraleae 15 g, *Achyranthes* root 15 g, peach seed 10 g, flowers carthami 10 g, Rhizoma Chuanxiong 10 g, herba Lycopi 10 g, liquorice 6 g, *Asarum* 3 g	Decoction	Hospital preparation
Song 2017 [56]	Duhuo Jisheng decoction	*Radix Angelicae Pubescentis* 15 g, parasitic *Loranthus* 15 g, glossy privet fruit 15 g, Semen Cuscutae 15 g, *Angelica sinensis* 15 g, *Eucommia ulmoides* 15 g, Barbary wolfberry fruit 15 g, *Radix Sileris* 15 g, Semen Psoraleae 15 g, *Achyranthes* root 15 g, peach seed 10 g, flowers carthami 10 g, Rhizoma Chuanxiong 10 g, herba Lycopi 10 g, liquorice 6 g, *Asarum* 3 g	Decoction	Hospital preparation
Sun 2018 [57]	Qinbi decoction	Chinese starjasmine stem 12 g, Caulis Sinomenii 12 g, honeysuckle stem 12 g, Semen Coicis 21 g, Tuckahoe 15 g, *Achyranthes* root 12 g, parasitic *Loranthus* 15 g, *Radix Stephaniae Tetrandrae* 9 g, *Cortex Phellodendri* 9 g, root of common peony 9 g, *Radix Clematidis* 9 g, parasitic *Loranthus* 9 g, *Rhizoma Corydalis* 6 g, liquorice 6 g	Decoction	Hospital preparation
Tan 2014 [58]	Huangqi Biejia pill	Ginseng, Cortex Cinnamomi, *Radix Rehmanniae Recen*, *Pinellia ternata*, *Radix Asteris*, *Rhizoma Anemarrhenae*, *Astragalus*, liquorice, *Radix Asparagi*, Carapax Trionycis, *Gentiana macrophylla*, white poria, Cortex Lycii Radicis, *Radix Bupleuri*	Pill	Hospital preparation
Tang 2012 [59]	Jiedu Yishen pill	*Radix Cynanchi Panicullati* 120 g, *Achyranthes* root 80 g, *Eucommia ulmoides* 80 g, Rhizoma Chuanxiong 80 g, woodlouse 40 g, Cortex Phellodendri 40 g	Pill	Hospital preparation
Wang 2013 [60]	Fufang Xiatian Wu pill	*Corydalis amabilis*, *Radix Aconiti Kusnezoffi Preparata*, herba *Siegesbeckiae*, *Cissus assamica*, Caulis Spatholobi, *Paederia scandens*, *Radix Clematidis*, *Aristolochia fangchi*, Cortex Acanthopanacis, *Rhizoma seu Radix Notopterygii*, *Gentiana macrophylla*, *Agkistrodon*, herba ephedra	Pill	SFDA approval number: Z20003105
Wang 2019 [61]	Taoren Xikang pill	Peach seed, flowers carthami, *Angelica sinensis*, *Radix Rehmanniae Praeparata*, Rhizoma Chuanxiong, *Radix Paeoniae Alba*, *Radix Angelicae Pubescentis*, *Radix Sileris*, parasitic *Loranthus*, *Achyranthes* root, *Asarum*, olibanum, myrrh	Pill	Henan FDA approval number: Z20120243
Wen 2016 [62]	Duhuo Jisheng decoction	*Radix Angelicae Pubescentis* 15 g, parasitic *Loranthus* 15 g, glossy privet fruit 15 g, Semen Cuscutae 15 g, *Angelica sinensis* 15 g, *Eucommia ulmoides* 15 g, Barbary wolfberry fruit 15 g, *Radix Sileris* 15 g, Semen Psoraleae 15 g, *Achyranthes* root 15 g, peach seed 10 g, flowers carthami 10 g, Rhizoma Chuanxiong 10 g, herba Lycopi 10 g, liquorice 6 g, *Asarum* 3 g	Decoction	Hospital preparation
Wu 2012 [65]	Zhuanggu Tongbi pill	*Radix Rehmanniae Praeparata* 12 g, *Eucommia ulmoides* 15 g, herba Pyrolae 30 g, pulp of dogwood fruit 15 g, Rhizoma Drynariae 15 g, *Radix Clematidis* 30 g, *Radix Dipsaci* 15 g, *Achyranthes* root 15 g, garden balsam stem 15 g, Chinese *Polyphaga* 9 g, Tuckahoe 12 g, *Radix Aconiti Preparata* 9 g, *Fructus psoraleae* 15 g, parasitic *Loranthus* 15 g	Pill	Hospital preparation
Wu 2018 [63]	Bushen Huoxue decoction	*Radix Rehmanniae Praeparata* 15 g, *Epimedium* 15 g, Semen Cuscutae 15 g, *Angelica sinensis* 10 g, *Salviae miltiorrhizae* 10 g, *Achyranthes* root 10 g, *Eucommia ulmoides* 10 g, *Radix Dipsaci* 10 g, *Radix Codonopsis* 15 g, *Rhizoma Atractylodis macrocephalae* 15 g, liquorice 6 g	Decoction	Hospital preparation
Wu 2018 [64]	Sanqi Xuejie capsule	*Notoginseng root Radix*, Resina Draconis	Capsule	Hospital preparation
Xia 2017 [66]	Jinwu Gutong capsule	Rhizoma Cibotii, *Epimedium*, *Radix Clematidis*, Zaocys dhumnade, *Achyranthes* root, Chinese quince, root of kudzu vine, *Radix Curcumae Longae*, *Fructus psoraleae*, *Radix Campanumoeae*	Capsule	SFDA approval number: Z20043621
Yang 2016 [67]	Jiawei Simiao Pulvis	Cortex Phellodendri 5 g, Rhizoma Atractylodis 10 g, Semen Coicis 10 g, *Achyranthes* root 10 g, *Radix Stephaniae Tetrandrae* 10 g, *Fructus forsythiae* 10 g, *Radix Sophorae Flavescentis* 10 g, Chinese quince 10 g, *Gentiana macrophylla* 10 g, *Radix Rehmanniae Recen* 15 g, honeysuckle stem 15 g	Pulvis	Hospital preparation
Yang 2019 [68]	Duhuo Jisheng decoction	*Radix Angelicae Pubescentis* 15 g, parasitic *Loranthus* 15 g, glossy privet fruit 15 g, Semen Cuscutae 15 g, *Angelica sinensis* 15 g, *Eucommia ulmoides* 15 g, Barbary wolfberry fruit 15 g, *Radix Sileris* 15 g, Semen Psoraleae 15 g, *Achyranthes* root 15 g, peach seed 10 g, flowers carthami 10 g, Rhizoma Chuanxiong 10 g, herba Lycopi 10 g, liquorice 6 g, *Asarum* 3 g	Decoction	Hospital preparation
Yao 2013 [69]	Duhuo Jisheng decoction	*Radix Angelicae Pubescentis* 15 g, parasitic *Loranthus* 15 g, glossy privet fruit 15 g, Semen Cuscutae 15 g, *Angelica sinensis* 15 g, *Eucommia ulmoides* 15 g, Barbary wolfberry fruit 15 g, *Radix Sileris* 15 g, Semen Psoraleae 15 g, *Achyranthes* root 15 g, peach seed 10 g, flowers carthami 10 g, Rhizoma Chuanxiong 10 g, herba Lycopi 10 g, liquorice 6 g, *Asarum* 3 g	Decoction	Hospital preparation
Yi 2017 [70]	Bushen Huoxue decoction	*Radix Rehmanniae Praeparata* 15 g, *Epimedium* 15 g, Semen Cuscutae 15 g, *Angelica sinensis* 10 g, *Salviae miltiorrhizae* 10 g, *Achyranthes* root 10 g, *Eucommia ulmoides* 10 g, *Radix Dipsaci* 10 g, *Radix Codonopsis* 15 g, *Rhizoma Atractylodis macrocephalae* 15 g, liquorice 6 g	Decoction	Hospital preparation
Yi 2017 [71]	Qiangjin Zhuanggu decoction	Semen Cuscutae 15 g, Rhizoma Curculiginis 20 g, *Morinda officinalis* 10 g, *Radix Rehmanniae Praeparata* 15 g, parasitic *Loranthus* 10 g, Radix Aconiti Preparata 9 g, Scorpio 4 g, centipede 4 g, Radix Clematidis 15 g, Lycopodium clavatum 15 g, Caulis Spatholobi 15 g, Rhizoma Chuanxiong 15 g, *Ramulus mori* 10 g, *Radix Sileris* 10 g, *Tribulus terrestris* 15 g, liquorice 10 g	Decoction	Hospital preparation
Yu 2010 [72]	Duhuo Jisheng decoction	*Radix Angelicae Pubescentis* 15 g, parasitic *Loranthus* 15 g, glossy privet fruit 15 g, Semen Cuscutae 15 g, *Angelica sinensis* 15 g, *Eucommia ulmoides* 15 g, Barbary wolfberry fruit 15 g, *Radix Sileris* 15 g, Semen Psoraleae 15 g, *Achyranthes* root 15 g, peach seed 10 g, flowers carthami 10 g, Rhizoma Chuanxiong 10 g, herba Lycopi 10 g, liquorice 6 g, *Asarum* 3 g	Decoction	Hospital preparation
Yuan 2017 [73]	Bushen Huoxue decoction	*Radix Rehmanniae Praeparata* 15 g, *Epimedium* 15 g, Semen Cuscutae 15 g, *Angelica sinensis* 10 g, *Salviae miltiorrhizae* 10 g, *Achyranthes* root 10 g, *Eucommia ulmoides* 10 g, *Radix Dipsaci* 10 g, *Radix Codonopsis* 15 g, *Rhizoma Atractylodis macrocephalae* 15 g, liquorice 6 g	Decoction	Hospital preparation
Zhang 2016 [74]	Jinwu Gutong capsule	Rhizoma Cibotii, *Epimedium*, Radix Clematidis, Zaocys dhumnade, *Achyranthes* root, Chinese quince, root of kudzu vine, Radix Curcumae Longae, *Fructus psoraleae*, Radix Campanumoeae	Capsule	SFDA approval number: Z20043621
Zhang 2017 [77]	Fugui Gutong capsule	Radix Aconiti Lateralis Preparata, Radix Aconiti Preparata, Cortex Cinnamomi, *Codonopsis pilosula*, *Angelica sinensis*, *Radix Paeoniae Alba*, *Epimedium*, olibanum	Capsule	SFDA approval number: Z19990026
Zhang 2018 [76]	Qianggu capsule	Rhizoma Drynariae 0.25 g	Capsule	SFDA approval number: Z20030007
Zhang 2019 [75]	Duhuo Jisheng decoction	*Radix Angelicae Pubescentis* 15 g, parasitic *Loranthus* 15 g, glossy privet fruit 15 g, Semen Cuscutae 15 g, *Angelica sinensis* 15 g, *Eucommia ulmoides* 15 g, Barbary wolfberry fruit 15 g, *Radix Sileris* 15 g, Semen Psoraleae 15 g, *Achyranthes* root 15 g, peach seed 10 g, flowers carthami 10 g, Rhizoma Chuanxiong 10 g, herba Lycopi 10 g, liquorice 6 g, *Asarum* 3 g	Decoction	Hospital preparation
Zheng 2014 [78]	Zhengqing Fengtongning tablet	Sinomenine 60 mg	Tablet	SFDA approval number: Z20010174
Zheng 2019 [79]	Bushen Huoxue decoction	*Radix Rehmanniae Praeparata* 15 g, *Epimedium* 15 g, Semen Cuscutae 15 g, *Angelica sinensis* 10 g, *Salviae miltiorrhizae* 10 g, *Achyranthes* root 10 g, *Eucommia ulmoides* 10 g, *Radix Dipsaci* 10 g, *Radix Codonopsis* 15 g, *Rhizoma Atractylodis macrocephalae* 15 g, liquorice 6 g	Decoction	Hospital preparation
Zhong 2017 [80]	Shentong Zhuyu decoction	*Angelica sinensis* 15 g, Rhizoma Chuanxiong 15 g, *Achyranthes* root 15 g, peach seed 12 g, flowers carthami 12 g, *Gentiana macrophylla* 12 g, *Rhizoma seu Radix Notopterygii* 12 g, Rhizoma Cyperi 9 g, myrrh 9 g, earthworm 9 g, Trogopterus Dung 6 g, liquorice 6 g	Decoction	Hospital preparation
Zhou 2012 [81]	Shufu Jiangu decoction	Radix Aconiti Lateralis Preparata 10 g, *Radix Rehmanniae Praeparata* 20 g, Rhizoma Drynariae 10 g, *Radix Dipsaci* 15 g, *Radix Angelicae Pubescentis* 10 g, *Achyranthes* root 15 g, Radix Clematidis 10 g, *Salviae miltiorrhizae* 15 g, pangolin scales 10 g, Scorpio 3 g, *Ramulus mori* 15 g, liquorice 6 g	Decoction	Hospital preparation
Zhu 2013 [82]	Zhengqing Fengtongning tablet	Sinomenine 60 mg	Tablet	SFDA approval number: Z20010174
Zhuo 2019 [83]	Rendong Bixie decoction	Honeysuckle stem 30 g, yam rhizome 15 g, Radix Clematidis 12 g, bark of Himalayan coralbean 15 g, root of common peony 12 g, Radix Gentianae Macrophyllae 15 g, herba Siegesbeckiae 15 g, loofah sponge 20 g, *Ramulus mori* 30 g	Decoction	Hospital preparation

SFDA: State Food and Drug Administration; FDA: Food and Drug Administration.

**Table 4:** Frequently used herbs in included studies.

Chinese name	Latin name	English name	Family	Number of studies (%)
Niu xi	*Radix Achyranthis Bidentatae*	Twotooth *Achyranthes* root	*Amaranthaceae*	33 (58.93%)
Di Huang	*Radix Rehmanniae*	Rehmannia root	*Scrophulariaceae*	30 (53.57%)
Dang Gui	*Radix Angelicae Sinensis*	Chinese angelica	*Apiaceae*	29 (51.79%)
Chuan Xiong	*Radix Ligustici Wallichii*	Sichuan lovage rhizome	*Apiaceae*	29 (51.79%)
Du Zhong	*Cortex Eucommiae Ulmoidis*	*Epimedium*	*Eucommiaceae*	26 (46.43%)
Gan Cao	*Radix Glycyrrhizae*	Liquorice	*Papilionaceae*	25 (44.64%)
Du Huo	*Radix Angelicae Pubescentis*	Pubescent angelica root	*Apiaceae*	21 (37.50%)
Sang Ji sheng	*Radix Loranthi Seu visci*	Chinese taxillus twig	*Loranthaceae*	21 (37.50%)
Bai shao	*Radix Paeoniae Alba*	White paeony root	*Asclepiadaceae*	19 (33.93%)
Fang Feng	*Radix Ledebouriellae Divaricatae*	Divaricate saposhnikovia root	*Apiaceae*	18 (32.14%)
Qin Jiao	*Radix Gentianae Macrophyllae*	Largeleaf gentian root	*Gentianaceae*	18 (32.14%)
Fu Ling	*Poria Cocos Wolff*	Tuckahoe	*Polyporaceae*	17 (30.36%)
Xi Xin	*Asari Radix et Rhizoma*	*Asarum*	*Aristolochiaceae*	16 (28.57%)
Yin Yang Huo	*Epimedium brevicornu Maxim*	Icariin	*Berberidaceae*	11 (19.64%)
Huang qi	*Radix Astragali Membranacei*	*Astragalus*	*Leguminosae*	11 (19.64%)
Wei Ling Xian	*Radix Clematidis Chinensis*	Chinese clematis root	*Ranunculaceae*	11 (19.64%)
Bu Gu Zhi	*Psoralea corylifolia Linn*	*Fructus psoraleae*	*Leguminosae*	11 (19.64%)

## Data Availability

Previously reported data were used to support this study. These prior studies and datasets are cited at relevant places within the text as references [28–83].

## Abbreviations

ADR: Adverse drug reactions
RCT: Randomized controlled trial
TG: Trial group
CG: Control group
COA: Chinese Orthopedic Association
CRA: Chinese Rheumatology Association
ACR: American College of Rheumatology
VAS: Visual analog score
WOMAC: Western Ontario and McMaster Universities Osteoarthritis Index
ER: Effective rate
NR: Not reported
CHM: Chinese herbal medicine
KOA: Knee osteoarthritis
NSAIDS: Nonsteroidal anti-inflammatory drugs
PRISMA: Preferred Reporting Items for Systematic Reviews and Meta-Analyses
WCM: Western conventional medication
TCM: Traditional Chinese medicine
SMD: Standard mean difference
RR: Relative risk
DJD: Duhuo Jisheng decoction
ASU: Avocado soybean unsaponifiables.

## Supplementary Materials

Supplementary Materials PubMed search strategies and graphical abstract were provided as supplementary material.
