# Supplementary material for: The Efficacy and Safety of Chinese Herbal Medicine in the Treatment of Knee Osteoarthritis: An Updated Systematic Review and Meta-Analysis of 56 Randomized Controlled Trials
Source: Oxid Med Cell Longev. 2022 Jan 7;2022:6887988. doi: 10.1155/2022/6887988 (PMC8759838; doi:10.1155/2022/6887988)
Supplement: Supplementary Materials — PubMed search strategies and graphical abstract were provided as supplementary material. [file 6887988.f1.zip › Graphic Abstract.pdf]

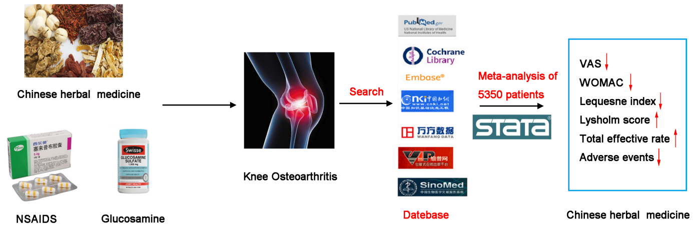
Chinese herbal medicine
NSAIDS
Glucosamine
Knee Osteoarthritis
Search
PubMed.gov
Cochrane Library
Embase®
万方数据
WANFANG DATA
SinoMed
Datebase
Meta-analysis of
5350 patients
STATA
VAS
WOMAC
Lequesne index
Lysholm score
Total effective rate
Adverse events
Chinese herbal medicine
