# Supplementary material for: The Efficacy and Safety of Chinese Herbal Medicine in the Treatment of Knee Osteoarthritis: An Updated Systematic Review and Meta-Analysis of 56 Randomized Controlled Trials
Source: Oxid Med Cell Longev. 2022 Jan 7;2022:6887988. doi: 10.1155/2022/6887988 (PMC8759838; doi:10.1155/2022/6887988)
Supplement: Supplementary Materials — PubMed search strategies and graphical abstract were provided as supplementary material. [file 6887988.f1.zip › PubMed search strategy.docx]

#1. Osteoarthritis [mh]

#2. Knee osteoarthritis [mh]

#3. Or/1-2

#4. Medicine, Chinese Traditional [mh]

#5. Herbal Medicine [mh]

#6. Integrative Medicine [mh]

#7. Traditional Chinese medicine [tiab]

#8. Herb∗[tiab]

#9. Or/3-8

#10. #3 and #9

#11. Randomized controlled trial [pt]

#12. Controlled clinical trial [pt]

#13. Randomized [tiab]

#14. Placebo [tiab]

#15. Drug therapy [sh]

#16. Randomly [tub]

#17. Groups [tub]

#18. Or/11–17

#19. Animals [mph] not (humans [min] and animals [min])

#20. 18 not 19

#21. #10 and #19
